# Interlocking host and viral *cis*-regulatory networks drive Merkel cell carcinoma

**DOI:** 10.1172/JCI188924

**Published:** 2025-12-15

**Authors:** Lingling Miao, David Milewski, Amy Coxon, Tara Gelb, Khalid A. Garman, Jadon Porch, Arushi Khanna, Loren Collado, Natasha T. Hill, Kenneth Daily, Serena Vilasi, Danielle Reed, Tiffany Alexander, Gabriel J. Starrett, Maharshi Chakraborty, Young Song, Rachel Choi, Vineela Gangalapudi, Josiah Seaman, Andrew Morton, Klaus J. Busam, Christopher R. Vakoc, Daniel J. Urban, Min Shen, Matthew D. Hall, Richard Sallari, Javed Khan, Berkley E. Gryder, Isaac Brownell

**Affiliations:** 1 Dermatology Branch, National Institute of Arthritis and Musculoskeletal and Skin Diseases (NIAMS), and; 2 Genetics Branch, National Cancer Institute (NCI), NIH, Bethesda, Maryland, USA.; 3 Department of Genetics and Genome Sciences, Case Western Reserve University, Cleveland, Ohio, USA.; 4 Laboratory of Cellular Oncology, NCI, NIH, Bethesda, Maryland, USA.; 5 Axiotl, Cleveland, Ohio, USA.; 6 Department of Pathology, Memorial Sloan Kettering Cancer Center, New York, New York, USA.; 7 Cold Spring Harbor Laboratory, Cold Spring Harbor, New York, USA.; 8 Division of Pre-clinical Innovation, National Center for Advancing Translational Sciences (NCATS), NIH, Rockville, Maryland, USA.

**Keywords:** Dermatology, Oncology, Epigenetics, Skin cancer

## Abstract

Over 15% of cancers worldwide are caused by viruses. Merkel cell polyomavirus (MCPyV) is the most recently discovered human oncovirus and is the only polyomavirus that drives malignant tumors in humans. Here, we show that MCPyV^+^ Merkel cell carcinoma is defined by neuroendocrine-lineage core regulatory (CR) transcription factors (TFs) (ATOH1, INSM1, ISL1, LHX3, POU4F3, and SOX2) that were essential for tumor survival and that co-bound chromatin with the viral small T antigen at super enhancers. Moreover, MCPyV integration sites were enriched at these neuroendocrine super enhancers. We further discovered that the MCPyV noncoding control region contained a homeodomain binding motif absent in other polyomaviruses that bound ISL1 and LHX3 and depended on them for T antigen expression. To therapeutically target the CR factors, we used histone deacetylase (HDAC) inhibitors to collapse the chromatin architecture and induce topological blurring of superenhancer loops, abrogating core TF expression and halting tumor growth. To our knowledge, our study presents the first example of oncogenic cross-regulation between viral and human epigenomic circuitry to generate interlocking and essential transcriptional feedback circuits that explain why MCPyV causes neuroendocrine cancer and represent a tumor dependency that can be targeted therapeutically.

## Abstract 2

The core regulatory circuitry of Merkel cell carcinoma cross-regulates integrated polyomavirus, converging on super enhancers and creating a vulnerability to HDAC inhibitors.

## Introduction

Most cancers arise from accrual of somatic DNA mutations, but some are due to introduced viral oncogenes (1, 2). Merkel cell carcinoma (MCC) is an aggressive neuroendocrine skin cancer driven by either genomic integration of the Merkel cell polyomavirus (virus-positive MCC [VP-MCC], 80% of cases) or UV-induced mutations in tumor suppressors or signaling oncoproteins (virus-negative MCC [VN-MCC], 20% of cases) (3, 4). MCCs resemble mechanosensory Merkel cells that reside in the epidermis, and both VP-MCC and VN-MCC tumors express genes associated with the neuroendocrine lineage. VP-MCC is driven by T antigens expressed from integrated Merkel cell polyomavirus (MCPyV). Why or how MCC adopts a neuroendocrine state and the role T antigens play in redefining cellular regulatory circuits remains largely unknown.

Here, we show that VP-MCC utilizes lineage-specific neuroendocrine transcription factors (TFs), including ATOH1, INSM1, ISL1, LHX3, POU4F3, and SOX2, that constitute the core regulatory circuits (CRC) of MCC. MCPyV small T antigen (ST) and host CR TFs co-bound VP-MCC superenhancers (SEs), while T antigen expression was directly regulated by LHX3 and ISL1, establishing an interlocking network between host CR circuitry and the oncovirus. Moreover, MCPyV integration sites were enriched near VP-MCC SEs, further suggesting a functional relationship between viral oncogenesis and core neuroendocrine circuitry.

SEs are *cis*-regulatory elements that reflect identity-defining gene commitments (5, 6). SEs are regulated by CR TFs expressed at levels many fold higher than the median (7). CR TFs are often regulated by SEs, creating densely interconnected loops of cross-regulated CR TFs known as CRCs (7). The high expression and self-reinforcing nature of CRCs are considered key factors in maintaining stable cell identity. We have previously shown that CR transcription is especially susceptible to histone deacetylase inhibitors (HDACi) (8). HDACi are generally expected to increase the transcription of genes due to the role of HDACs as erasers of active histone acetylation. Counterintuitively, HDACi result in a highly specific and robust decrease in the transcription of SE-driven CR TFs in rhabdomyosarcoma and other contexts (8, 9). Here, we show that the core circuitry of VP-MCC can also be selectively depleted after HDACi treatment. HDACi treatment of VP-MCC xenografts in mice led to greatly decreased tumor volume and prolonged survival but showed no response in VN-MCC xenografts. Through the use of absolute quantification of architecture (AQuA) HiChIP, we demonstrate that HDACi repression of SE-driven CR TFs was mediated by a phenomenon we refer to as “topological blurring,” whereby the chromatin contact frequencies between enhancers and CR TF promoters decreased in spite of a general increase in acetylation.

## Results

### Neuroendocrine CR circuits dominate VP-MCC.

**Figure 1:**
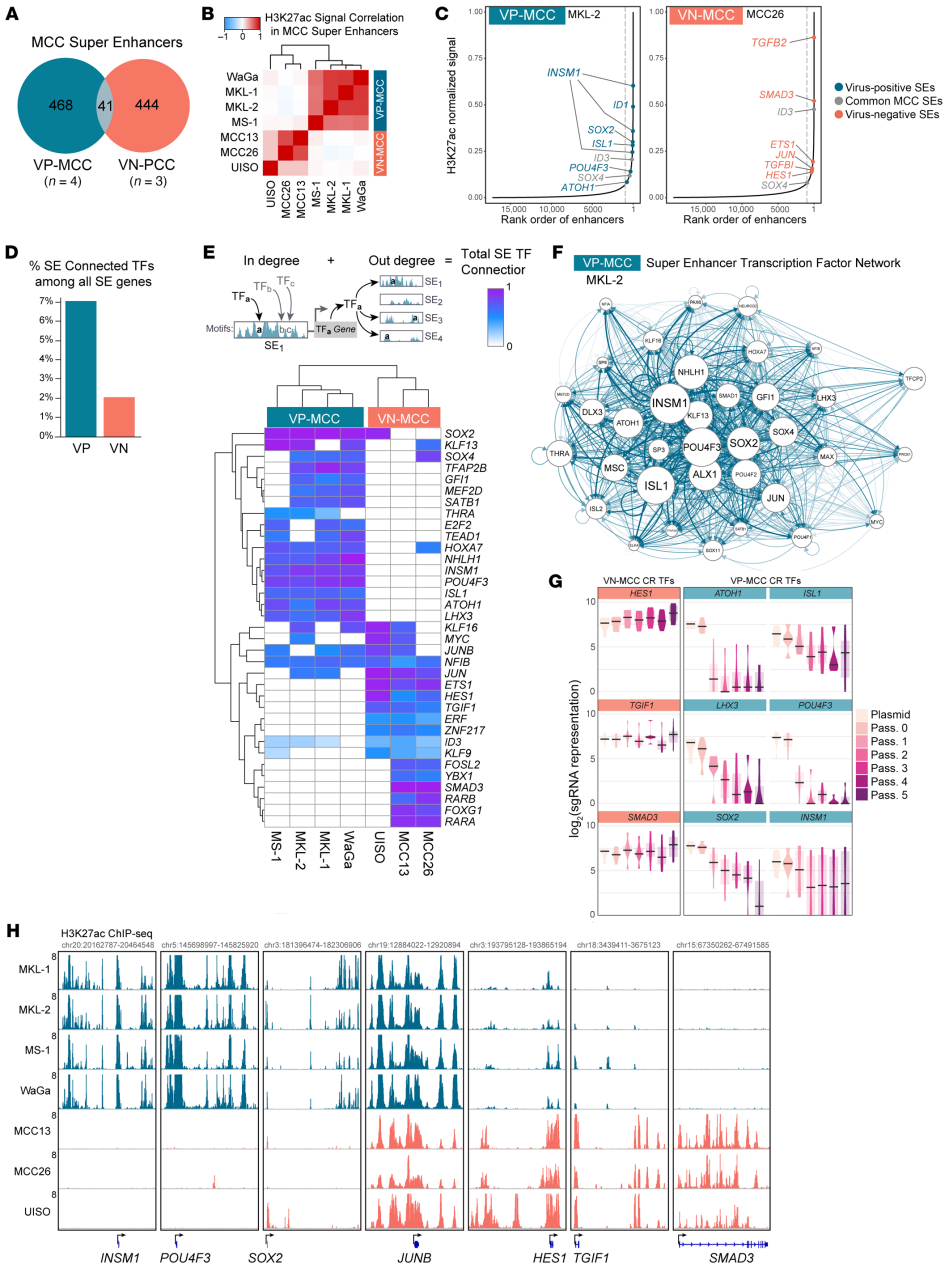
Neuroendocrine CR TF network in VP-MCC. (**A**) SEs common to 4 VP-MCC and 3 VN-MCC cell lines and overlap. (**B**) SE clusters by Pearson correlation of H3K27ac signal at all MCC SEs. (**C**) Representative rank order of SE (ROSE) plots. (**D**) Proportion of SEs connected to TF genes in VP-MCC (7%) and VN-MCC (2%). (**E**) CRC prediction by analysis of SE-associated TFs. Degree of connectivity for each TF is the combination of the “in” degree (the number of all TF motifs in its own SE) and the “out” degree (the total number of other SEs with that TF’s motif present), summarized in a heatmap. (**F**) Network of CR TFs in VP-MCC. Each arrow represents the presence of a TF’s motif in the SE of the TF to which it is pointing. Node sizes are scaled by relative gene expression. (**G**) Box plot violin representation of sgRNA abundance in MKL-1 cells over sequential passages, showing dropout of CR TFs co-opted in VP-MCC. (**H**) Genome browser view of H3K27ac ChIP-Seq at select CR TFs in MCC.

To determine active enhancers in MCC, we performed ChIP-Seq of acetylated histone H3 at lysine 27 (H3K27ac) in 4 VP-MCC and 3 VN-MCC cell lines. SEs were identified as having the most concentrated deposits of H3K27ac. Consistent with their disparate pathoetiologies and possibly distinct cells of origin (3, 4), very few (8.8%) VP-MCC SEs overlapped with VN-MCC SEs (Figure 1, A and B, and Supplemental Figure 1A; supplemental material available online with this article; https://doi.org/10.1172/JCI188924DS1). In VN-MCC, we observed an enrichment of SEs associated with genes related to cell signaling pathways, especially TGF-β (Figure 1, C and D, and Supplemental Figure 1, B and C), consistent with aberrant signaling from reported genetic alterations of *RB1*, *TP53*, *PIK3CA*, and *NOTCH1/2* (10, 11). Genes associated with SEs in VP-MCC cells were enriched in TFs, especially those related to the neuroendocrine lineage (Figure 1, C and D, and Supplemental Figure 1, B and C). SEs that regulate neurogenesis genes and TFs were H3K27ac abundant in VP-MCC cells and corresponded with higher gene expression, whereas a reciprocal pattern was present in VN-MCC cells (Supplemental Figure 1, C and D). Overall, 7% of SEs in VP-MCC targeted TFs compared with only 2% in VN-MCC (Figure 1D).

RNA-Seq revealed a set of TFs highly and selectively expressed in VP-MCC (Supplemental Figure 2 and Supplemental Table 1), many of which are associated with SEs specific to VP-MCC (Figure 1C and Supplemental Tables 1 and 2). To reconstruct the CR structure of MCC (Figure 1E), we analyzed the TF motifs present in TF SEs (5). We discovered interconnectivity among VP-MCC–specific TFs (Figure 1F), with ATOH1, INSM1, ISL1, LHX3, POU4F3, and SOX2 motifs present in SEs associated with each of these factors. To evaluate the necessity of CR TFs in VP-MCC, we performed pooled CRISPR disruption (library with 6–8 sgRNAs targeted to the DNA binding domains of each human TF) in MKL-1 cells, monitored over 5 cell passages. Remarkably, the CR TFs were each required in VP-MCC and dropped out at varying speeds (Figure 1G). Expression levels of these top 6 CR TFs in VP-MCC cells exceeded that of the most highly expressed CR TF in VN-MCC, *MYC* (Supplemental Figure 2D and Supplemental Table 1). When considering CR TFs’ gene expression profiles, VP-MCC cell lines matched that of tumors from patients with MCC (Supplemental Figure 2E) and harbored extensive enhancer structures at their CR TF loci (Figure 1H).

Consistent with a role in defining VP-MCC cell lineage, the top CR TFs function normally in sensory and neuroendocrine cell types. Merkel cells and sensory hair cells of the inner ear are innervated mechanoreceptors, and both require ATOH1 and SOX2 for proper development (12). SOX2 and ISL1 coregulate *Atoh1* expression in Merkel cell development (13). *Pou4f3* expression by inner ear hair cells is regulated by an enhancer activated by ATOH1 binding (14), and it is required for normal MC lineage development (15). INSM1 is a critical regulator of neuroendocrine cell development (16) and is a diagnostic immunohistochemical marker for neuroendocrine tumors (17). ISL1 and LHX3 are LIM homeobox TFs that regulate their own enhancers during neural development (18). Thus, similar to other neuroendocrine cancers (19), oncogenic transformation of VP-MCC involves TFs related to neural and neuroendocrine lineage specification.

**Figure 2:**
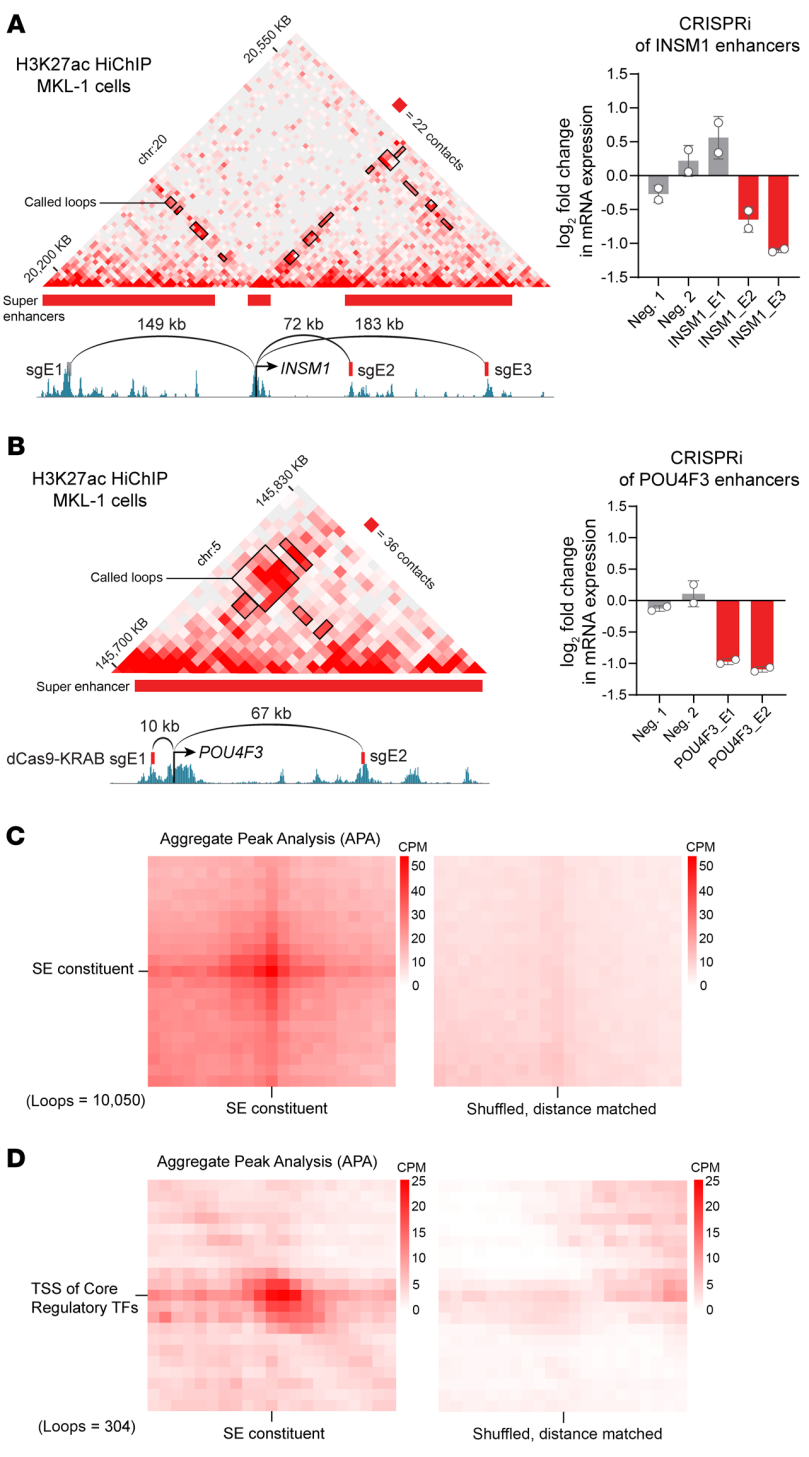
SEs loop to regulate CR TF genes in VP-MCC. (**A** and **B**) Left panels: Contact matrix of H3K27ac-mediated interactions (measured by H3K27ac HiChIP in MKL-1 cells), annotated with ChIP-Seq of H3K27ac, the genomic locations of sgRNAs targeting enhancer elements, and the CR TF gene. SEs are indicated with red bars, and called loops are shown with black rectangles. Right panels: *INSM1* (**A**) and *POU4F3* (**B**) mRNA expression in MKL-1 cells after CRISPi with sgRNAs targeting INSM1 and POU4F3 SEs, assayed by RT-qPCR. Neg., negative control sgRNAs. Distances between enhancer constituents and the CR TF target gene are indicated above the arcs connecting to the enhancer sgRNAs used to guide dCas9-KRAB for enhancer silencing. (**C**) APA plots of H3K27ac HiChIP data in MKL-1 cells, centered around SE loops (left) or 1 SE loop and a distance-matched shuffled location as a background control (right). (**D**) APA plots of H3K27ac HiChIP data in MKL-1 cells, centered around loops connecting SE constituents and their target TSS/promoter for CR TF genes.

To test whether the H3K27ac-rich enhancers were looping to the candidate promoters of CR TF genes, we performed chromatin conformation capture paired with H3K27ac IP (H3K27ac HiChIP). For each CR TF, we found interactions between enhancers and promoters and also between adjacent enhancers in these regions (Figure 2, A–D, and Supplemental Figure 3, A and B). To test the functionality of these acetylation clusters, we used CRISPR interference (CRISPRi) to repress SEs of the CR TF genes *INSM1* and *POU4F3* in VP-MCC cell lines. After 48 hours, repression of these SEs markedly reduced the expression of the target CR TFs, confirming SE-gene interactions as positive transcriptional units (Figure 2, A and B). SE loop constituents were well connected in the HiChIP data, both to each other (Figure 2C) and to their target CR TF genes (Figure 2D).

### MCPyV integration sites are proximal to VP-MCC SEs.

**Figure 3:**
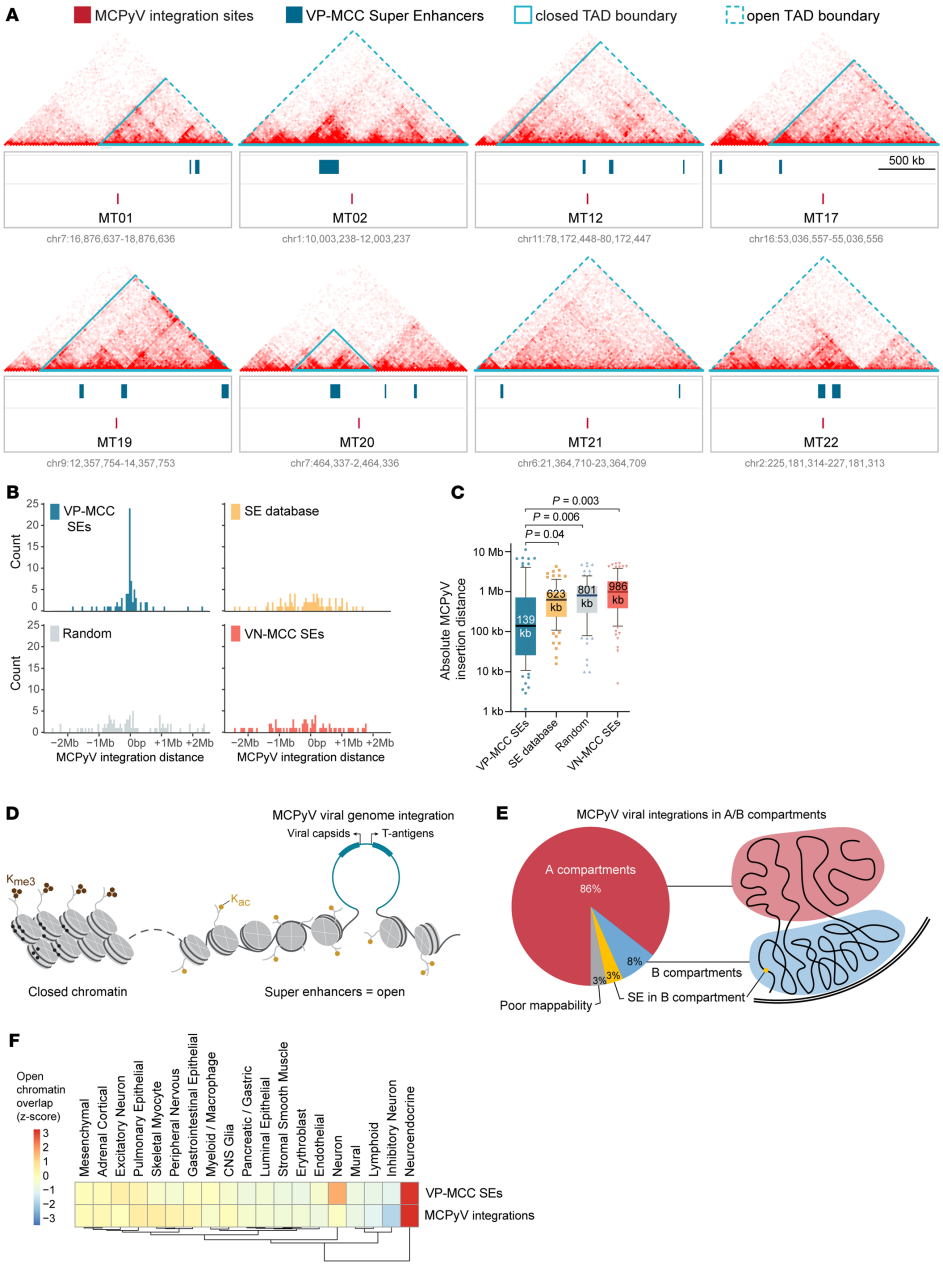
VP-MCC SEs are associated with MCPyV integration sites. (**A**) MCPyV integration sites (red) overlaid with SE sites that are recurrent among the 4 VP-MCC cell lines (sea blue). The clonal integration coordinates (unique for each VP-MCC tumor) are indicated in the center, and a 2 Mb window is shown around each. H3K27ac HiChIP from MKL-1 cells is shown above, with topological domain boundaries outlined in blue. (**B**) Histogram of the distances between MCPyV integration sites (*n* = 90 tumors) to the nearest VP-MCC SE, compared with SEs in other cell types, random genomic locations, and VN-MCC SEs. (**C**) Absolute distance between MCPyV integration sites and VP-MCC SEs, or to SEs from other human cell types and tissues, or to the nearest random genomic location, or to VN-MCC SEs. *P* values were determined using a Wilcoxon matched-pairs rank test. Data are summarized by box (quartiles) and whisker (1.5 × IQR) plots, with the median (thick center line). (**D**) Illustration of increased chromatin accessibility at SEs correlating with viral integration sites. (**E**) Percentage of integration sites by chromatin compartments defined by H3K27ac HiChIP data in MKL-1 cells. A compartment = euchromatin; B compartment = heterochromatin. (**F**) Heatmap of the human chromatin accessibility atlas (22) overlapped with VP-MCC SEs and MCPyV integration sites.

We identified the clonal polyomavirus integration sites for 11 newly characterized VP-MCC tumors, and 8 of 11 (72.7%) were found near — sometimes overlapping, and often within 1 Mb of — VP-MCC SEs (Figure 3A). Furthermore, integration sites in 8 of 13 (61.5%) VP-MCC cell lines and tumors (20) coincided with VP-MCC SEs, and 6 of 13 (46.1%) were near CR TF genes, including loci regulating *INSM1*, *ISL1*, and *LHX3* (Supplemental Figure 4), suggesting that these genes could have been active at the time of, or were activated by, MCPyV integration. To validate this observation, we analyzed MCPyV integration sites across 90 VP-MCC tumors (the above 24 cases and 66 additional published integration sites) and found the median distance to a VP-MCC SE was significantly smaller than the distance to the closest VN-MCC SE, or to random subsets of SEs in a database of human SEs (21) or the nearest random genomic location (Figure 3, B and C). The size of SEs was similar across subtypes, with VP-MCCs covering 1.3% of the genome, VN-MCCs covering 1.4% of the genome, and the control subsets each covering 1.3% of the genome. This preferential integration of MCPyV near VP-MCC SEs suggests a functional relationship between viral integration and CRC in the MCC cell of origin (Figure 3D). Consistent with MCPyV integration enrichment in active euchromatin near SEs, we found 85% of integration sites within the A compartment (defined by MKL-1 cell HiChIP), 3% in a SE found within the B compartment, and 8% in the B compartment with no SE nearby (Figure 3E). Comparing VP-MCC SEs and MCPyV integration sites with an atlas of chromatin accessibility in human tissue and cell types (22), we found the greatest overlap with chromatin sites active in neuroendocrine cell types (Figure 3F), further suggesting a positive association between transforming MCPyV integration events and neuroendocrine lineage circuitry.

### Neuroendocrine TFs are co-bound with ST at SEs.

**Figure 4:**
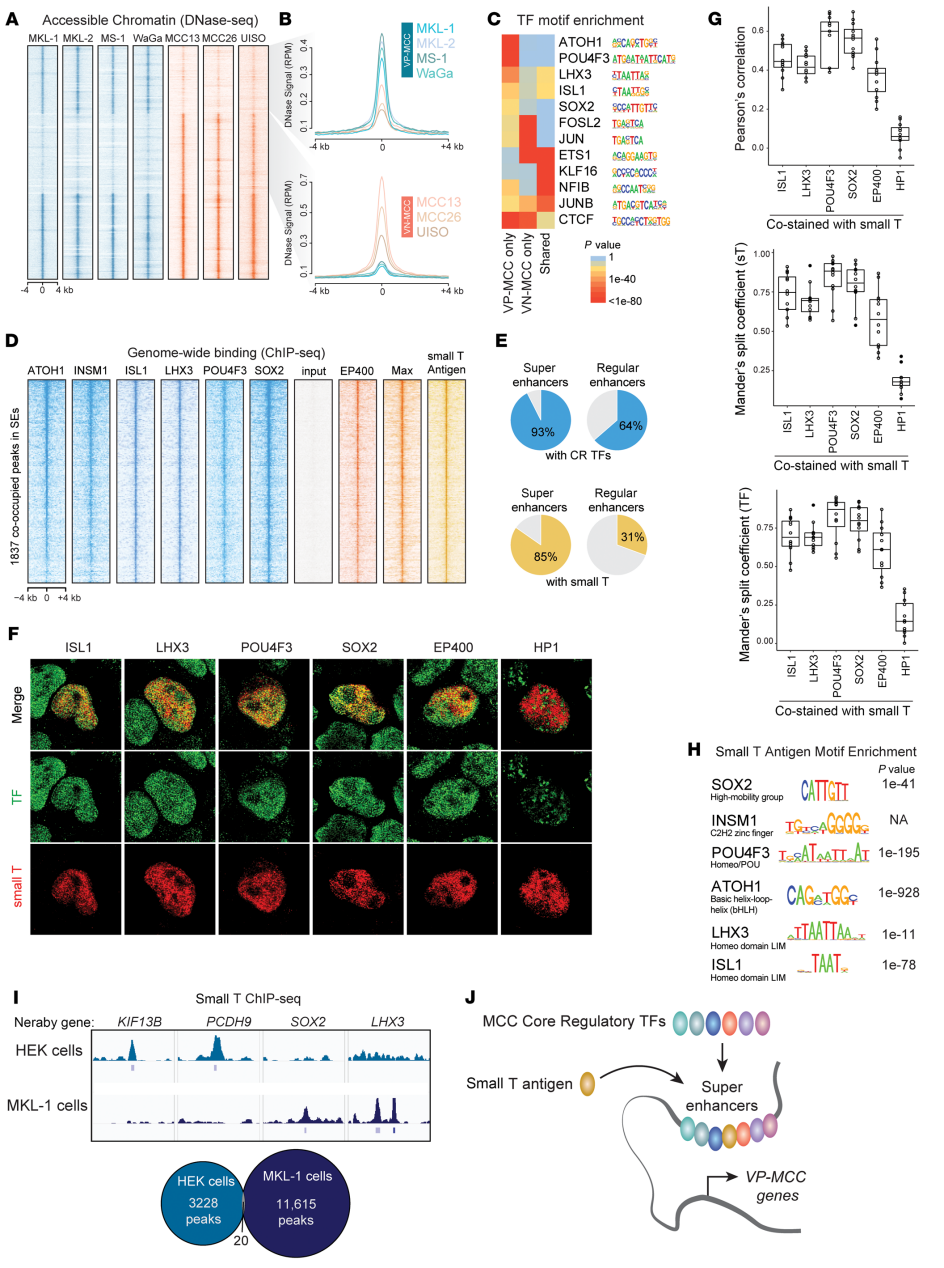
ATOH1, INSM1, ISL1, LHX3, POU4F3, and SOX2 co-bind SEs with MCPyV ST. (**A**) Heatmap of DNase hypersensitivity reveals sites of chromatin uniquely accessible in VP-MCC or VN-MCC. (**B**) DNase-Seq signal at sites with increased accessibility in VP-MCC (top) or VN-MCC (bottom). (**C**) TF motif enrichment within DNase hypersensitive sites predicts virus status–selective binding of TFs, plotted as a heatmap of *P* values determined by Hypergeometric Optimization of Motif EnRichment (HOMER) analysis. (**D**) Heatmap of ChIP-Seq at 1837 TF binding sites (±4 kb) within SEs in the VP-MCC cell line MKL-1. (**E**) Percentage of VP-MCC SEs and regular enhancers (in MKL-1 and MKL-2 cells) bound by CR TFs and ST. (**F**) Immunostaining of HA tag and CR TFs in the VP-MCC cell line MKL-1 transfected with HA-tagged ST. Original magnification, ×100. (**G**) Spatial correlation of nuclear ST and CR TF signals from immunostaining in **F**, as indicated by Pearson’s correlation and Mander’s split coefficients. (**H**) VP-MCC CR TF motifs enriched at genomic sites bound by the ST. (**I**) ChIP-Seq of ST transfected in HEK293 cells shows a lack of ST occupancy at VP-MCC sites. (**J**) Model of the VP-MCC CR network of neuroendocrine TFs and ST at SEs.

We hypothesized that access to open chromatin would be MCC viral subtype specific because of their distinct SE profiles and TF signatures. Indeed, DNase hypersensitivity in 4 VP-MCC and 3 VN-MCC cell lines showed that VP-MCC cells had shared sites of open chromatin, which were absent in VN-MCC cells (Figure 4, A and B), that were enriched in CR TF motifs including ATOH1, ISL1, LHX3, POU4F3, and SOX2 (Figure 4C). To validate the inferred regulatory network in VP-MCC, we performed ChIP-Seq for ATOH1, INSM1, ISL1, LHX3, POU4F3, and SOX2 in both MKL-1 and MKL-2 cells. Binding of these CR TFs was preferentially enriched in VP-MCC SEs, in which most SEs exhibited multiple peaks per TF (Figure 4, D and E, and Supplemental Figure 5, A and B).

In VP-MCC, the MCPyV oncogene ST binds MYCL, MAX, and the EP400 chromatin remodeling complex to promote gene expression that supports cellular proliferation, represses adhesion, and activates both neural and cutaneous developmental programs (23). Cross-analysis with ChIP-Seq targets for EP400, MAX, and ST in VP-MCC MKL-1 cells (23) revealed substantial overlap with targets bound by CR TFs in SEs (Figure 4, D and E, and Supplemental Figure 5A). VP-MCC SEs harbored ST and CR TF sites more frequently than did regular enhancers (Figure 4E), and ST binding was positively correlated with that of the CR TFs (Supplemental Figure 5, C and D). In VP-MCC, transfected or endogenous ST co-bound chromatin with various combinations of CR TFs (Supplemental Figure 5E), and overlap was far more frequent in SEs compared with the transcription start sites (TSSs) or promoters of active genes (Supplemental Figure 5F). CR TFs and ST also co-bound with MAX, a proto-oncogene and surrogate for its heterodimeric partner, MYCL. VP-MCC had high expression and unique SEs for *MYCL*; in contrast, VN-MCC had SEs driving *MYC* (Supplemental Figure 6). As detected by superresolution immunofluorescence microscopy, HA-tagged MCPyV small T antigen (HA-ST) overlapped with its known binding partner, EP400, as well as individual CR TFs in the euchromatin of HA-ST–transfected MKL-1 cells (Figure 4, F and G), confirming that ST co-occupies active chromatin compartments with VP-MCC CR TFs. Although ST does not have its own cognate DNA sequence, its ChIP peaks were enriched for neuroendocrine TF DNA motifs (Figure 4H). In support of a role for CR TFs in directing the localization of ST to VP-MCC SEs, ChIP-Seq of transfected ST in non-neuroendocrine HEK293 cells showed a lack of ST occupancy at VP-MCC SE loci (Figure 4I). Together, our data suggest a model in which ST is directed to VP-MCC SEs by the combined DNA specificities of these lineage-restricted CR TFs (Figure 4J).

### ISL1 and LHX3 bind a regulatory sequence unique to MCPyV to drive T antigen expression.

**Figure 5:**
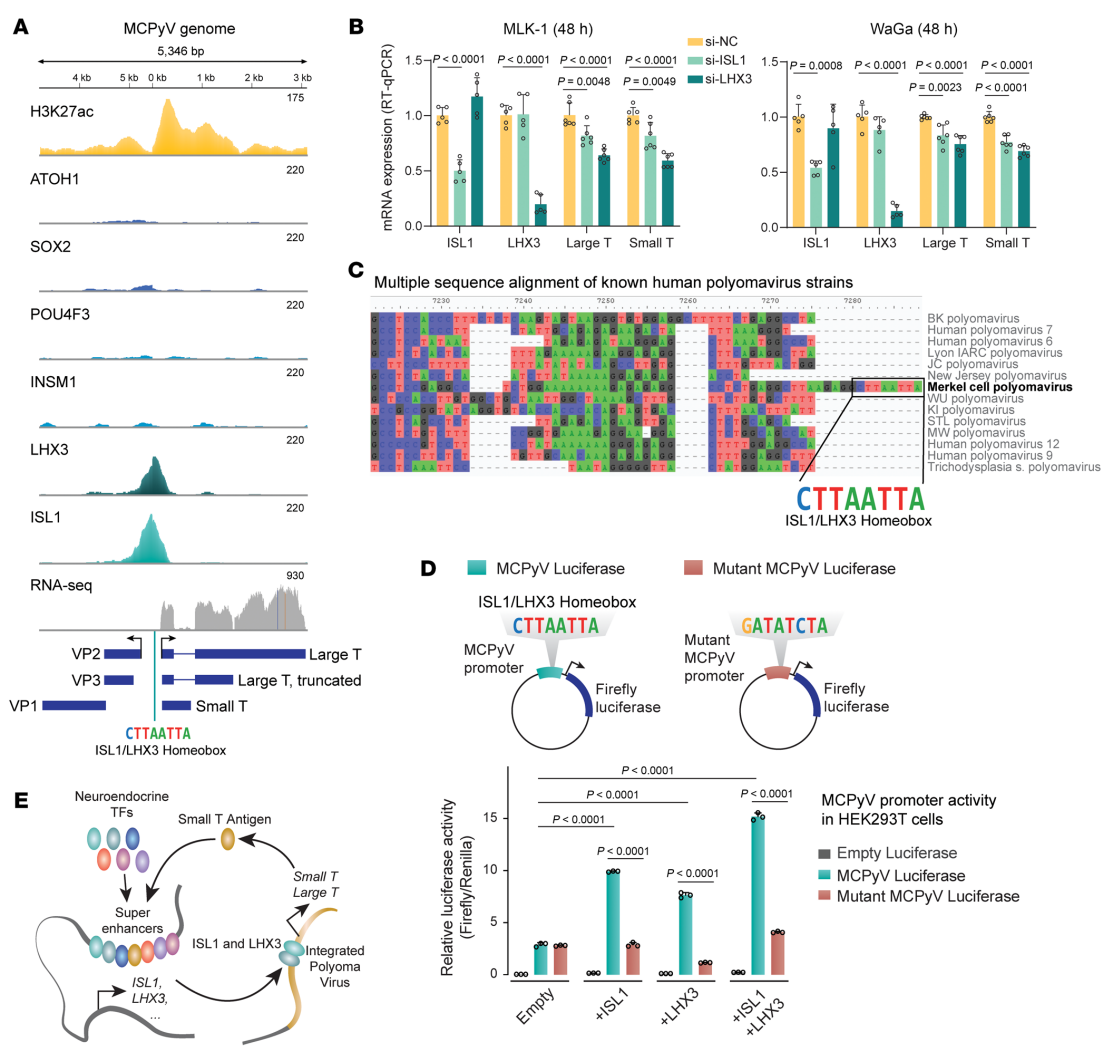
ISL1 and LHX3 bind and drive expression of polyomavirus T antigens. (**A**) MKL-1 ChIP-Seq of H3K27ac and CR TFs mapped to the integrated MCPyV genome reveals regulatory binding of ISL1 and LHX3, with RNA-Seq confirming expression of T antigens. (**B**) Knockdown of LHX3 or ISL1 caused downregulation of ST and LY, assayed by RT-qPCR in MKL-1 and WaGa cells. NC, nontargeting control. *P* values were calculated by 1-way ANOVA. Data are expressed as mean ± SD. (**C**) Multiple sequence alignment of human polyomavirus strains. The MCPyV unique ISL1/LHX3 homeobox sequence is highlighted. (**D**) Twenty-four-hour cotransfection of cDNA expression plasmids with WT or mutant MCPyV firefly and *Renilla* luciferase reporters in HEK293T cells. Values report the mean ± SD (*n* = 3 independent experiments) of the relative luciferase activity (firefly/*Renilla*). *P* values were calculated by 1-way ANOVA. (**E**) Model of interlocking polyomavirus and neuroendocrine TF cross-regulation circuit in VP-MCC.

In normal skin, MCPyV infection is common but T antigen expression is undetectable (24), whereas VP-MCC is rare and shows robust T antigen expression (25). We hypothesized that high T antigen expression in cancer cells may require the neuroendocrine CR TFs we identified, creating positive feedback circuitry between transforming viral oncogenes and a neuroendocrine state. Notably, ChIP-Seq reads mapped onto the noncoding control region of the polyomavirus genome indicated that the promoter driving MCPyV T antigens was flanked by H3K27ac and contained ISL1 and LHX3 peaks corresponding to an ISL1/LHX3 homeodomain motif (Figure 5A). ISL1 and LHX3 form a heterodimer when coexpressed (26). Knockdown of ISL1 or LHX3 in VP-MCC cells reduced the expression of both large T antigen (LT) and ST transcripts (Figure 5B). Aligning the promoter sequences of all known human polyomaviruses (HPyVs) revealed that this ISL1/LHX3 homeodomain motif is only present in MCPyV, and no other HPyVs (Figure 5C). To test this homeodomain motif, we designed a luciferase reporter plasmid containing a WT MCPyV promoter; it had strong activity in HEK293T cells cotransfected with ISL1 and/or LHX3 and reporter plasmids, which was reduced by mutating the homeodomain motif (Figure 5D). These data support a model of interlocking between the MCPyV oncogenes and the neuroendocrine-centered CR network found in VP-MCC (Figure 5E).

### Topological blurring via HDACi disrupts VP-MCC CR circuitry.

**Figure 6:**
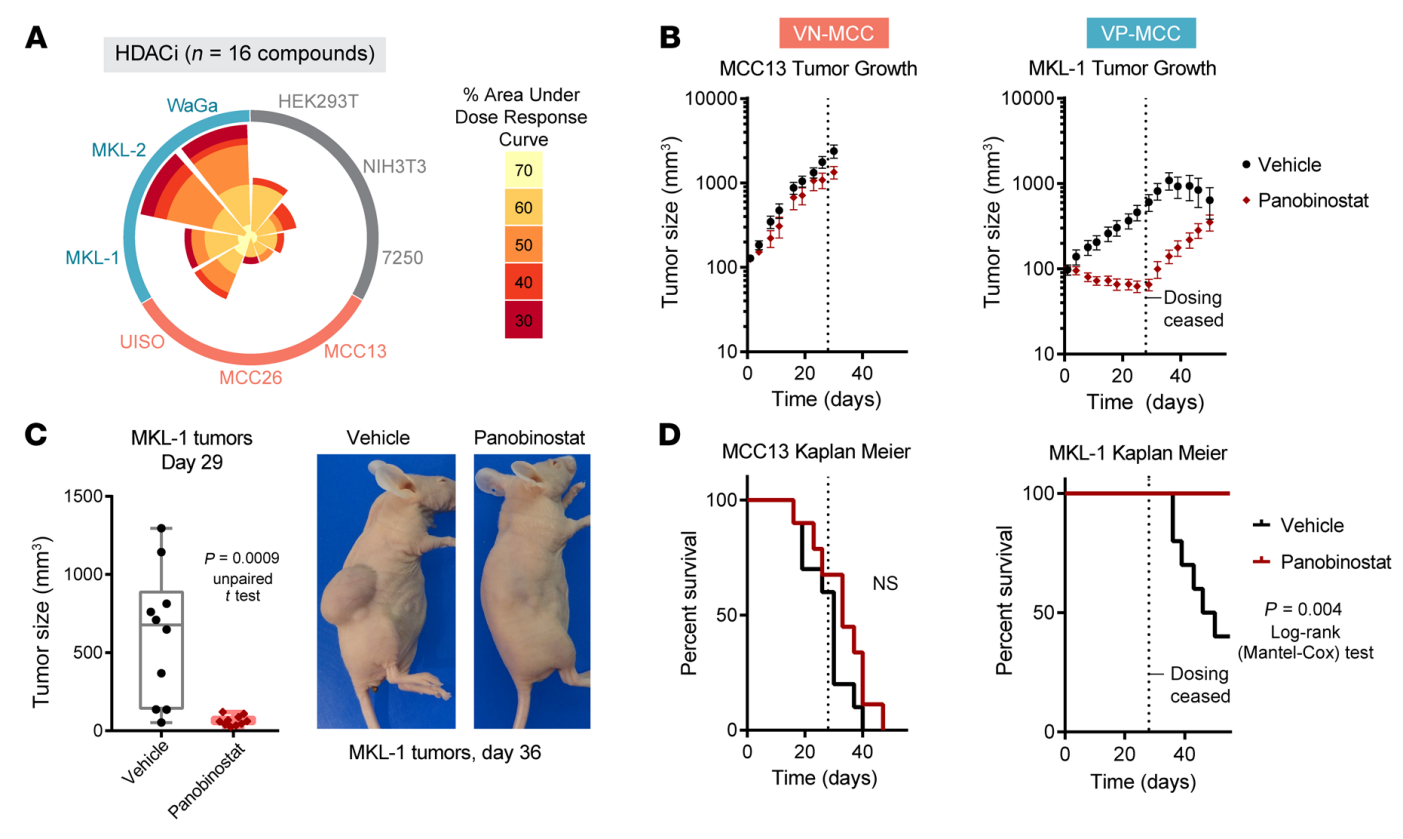
HDAC inhibition reduces VP-MCC tumor growth. (**A**) Wind-rose plot of AUCs for all HDACi (*n* = 16). Each petal shows the number of molecules that scored per cell line, with the color indicating the score magnitude. (**B**) Tumor growth curves for xenografts of MCC13 (VN-MCC) or MKL-1 (VP-MCC) treated with vehicle or HDACi panobinostat for 29 days. (**C**) Left: Box plot showing tumor sizes on day 29 of MKL-1 tumors, all data points overlapping. The *P* value calculated with an unpaired, 2-sided *t* test, box plots indicate first quartile, median, and third quartile, with whiskers at the minimum and maximum values. Right: Representative MKL-1 tumor–bearing mice treated with vehicle or panobinostat (day 36). (**D**) Kaplan-Meier plot for mice with MCC13 or MKL-1 xenografts treated with vehicle or panobinostat.

To test if CR TF addiction in VP-MCC would result in molecular vulnerabilities, we treated MCC cell lines with small-molecule HDACi, as CR transcription is especially susceptible to histone hyperacetylation (8). Multiple HDACi were more effective in VP-MCC (MKL-1, MKL-2, and WaGa) than in VN-MCC (MCC13, MCC26, and UISO) or control cell lines (HEK293T, NIH3T3, and 7250) (Figure 6A and Supplemental Figure 7). We further tested the clinically approved HDACi panobinostat, which was effective in all MCC cell lines tested but was more potent in VP-MCC cell lines (Supplemental Figure 7) in xenograft mouse models. The MCC13 (VN-MCC) tumors did not respond to panobinostat, but VP-MCC MKL-1 tumors greatly decreased in volume (Figure 6, B and C), resulting in prolonged survival (Figure 6D). As CR TFs can rely on HDACs for transcriptional output (8, 27), we performed RNA-Seq at 1 and 6 hours of panobinostat treatment in MKL-1 (VP-MCC) and MCC26 (VN-MCC) cells. In MKL-1 cells, we observed rapid and selective downregulation of VP-MCC neuroendocrine CR TFs (Supplemental Figure 8, A–C), whereas VN-MCC CR circuitry was unaffected, and antigen-processing genes were upregulated in both (Supplemental Figure 8, C and D), consistent with previous findings (28). Thus, the use of HDACi represents a strategy to target neuroendocrine TF addiction, kill tumor cells, and increase antigen presentation — a combination that could synergize with immunotherapy, the current standard of care for advanced MCC (3, 4).

**Figure 7:**
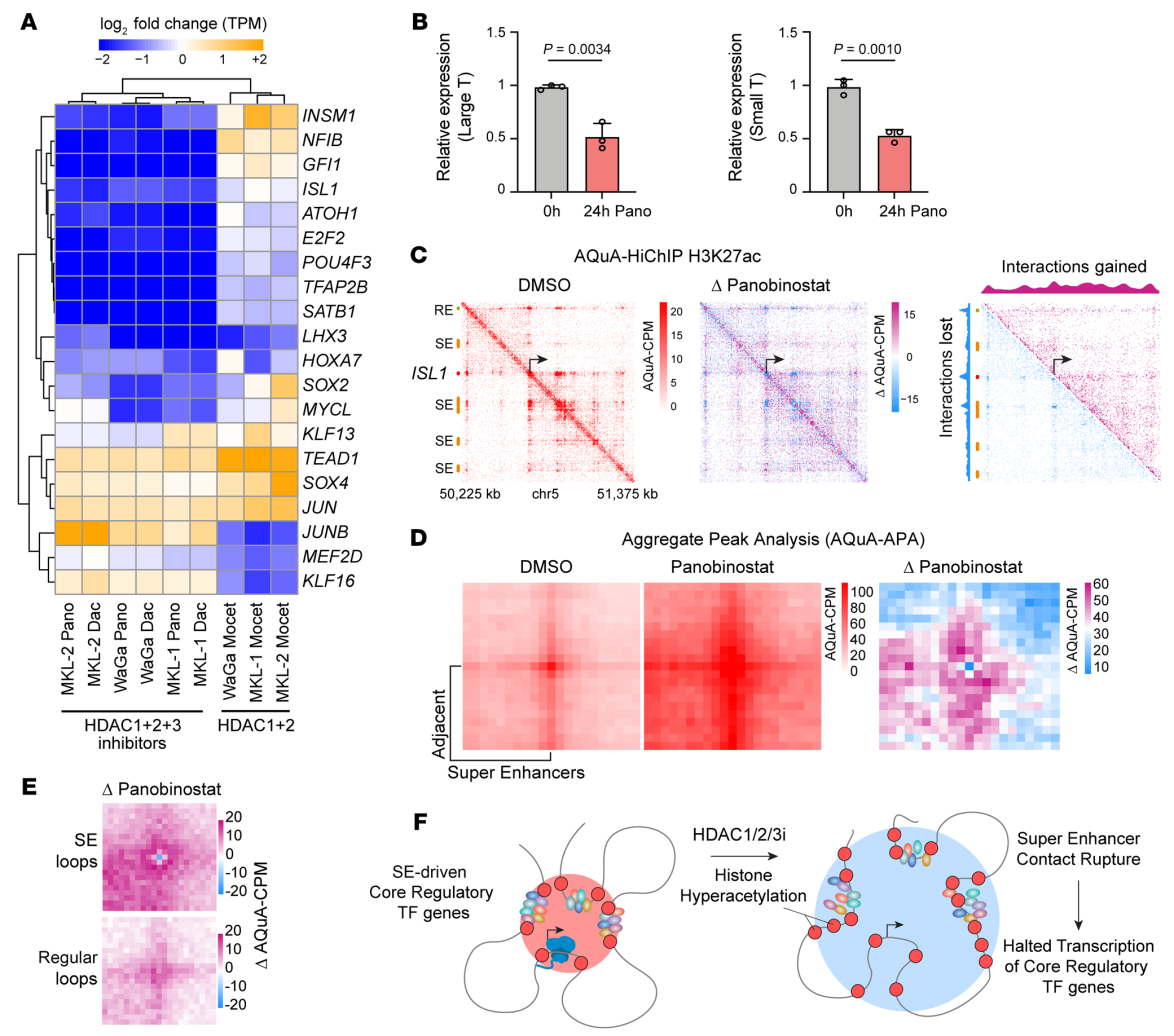
HDAC inhibition disrupts 3D enhancer loops to shutdown CR TFs. (**A**) Heatmap of expression changes for CR TFs (log_2_ fold change in TPM) in VP-MCC cell lines after treatment with panobinostat (Pano), dacinostat (Dac), or mocetinostat (Mocet). (**B**) RT-qPCR of MKL-1 cells shows decreased ST and LT expression following 24-hour panobinostat treatment. (**C**) AQuA-HiChIP of H3K27ac, performed after 6 hours of panobinostat treatment in WaGa cells, shows topological blurring in a heatmap of valid contacts per million reference contacts (AQuA-CPM) at the enhancer elements surrounding the *ISL1* gene. DMSO (left) and panobinostat (center) were normalized to exogenous mouse contacts and compared by subtraction of the treated and control contact matrices (right). Orange bars, SEs; red bar, *ISL1* gene. (**D**) APA plots for all adjacent SE pairs, AQuA normalized, in WaGa cells treated with DMSO (left) or panobinostat (middle) and contrasted by calculating the Δ (right). (**E**) AQuA-APA plot of called 3D loops in WaGa cells and the change induced at these loops by 6 hours of panobinostat treatment at SE loops or regular enhancer loops (bottom). (**F**) Model of H3K27ac diffusion and destabilization of long-range contacts by HDACi at CR TF SEs, followed by loss of CR TF gene expression.

We previously reported that the nuclear HDACs (HDAC1, HDAC2 and HDAC3) were co-essential in maintaining CR TF circuits in rhabdomyosarcoma (27). Guided by published HDAC isoform selectivity data from diverse HDACi (29), we chose panobinostat, dacinostat, and mocetinostat to evaluate the dependence of CR TF transcripts on HDACs. Each compound exhibited potency in HDAC1 and HDAC2, whereas dacinostat and panobinostat also inhibited HDAC3 (Supplemental Figure 8E). Upon a 6-hour treatment of 3 VP-MCC cell lines (MKL-1, MKL-2, and WaGa), rapid and selective downregulation of VP-MCC CR TFs was evident in all 3 cell lines upon treatment with the HDAC1/2/3 inhibitors panobinostat and dacinostat, while mocetinostat had a mild and mixed effect on the transcription of CR TFs (Figure 7A and Supplemental Figure 8, F, H, and I). Notably, the top CR TFs identified herein (*LHX3*, *ISL1*, *POU4F3*, *SOX2*, *INSM1*, *ATOH1*) were uniformly disrupted by panobinostat and dacinostat. Increased transcription of the p53 and apoptosis pathways (Supplemental Figure 8F) indicates mechanisms for the associated reductions in viability, which is consistent with these VP-MCC cell lines having intact p53 (30). Consistent with this finding, panobinostat reduced MKL-1 cell viability and induced cell apoptosis 24 hours after treatment but had no significant effect at 6 hours (Supplemental Figure 9). HDACi did not cause an acute drop in the expression of MCPyV T antigens at 6 hours (Supplemental Figure 8G). However, we found that panobinostat eventually decreased LT and ST expression 24 hours after treatment in MKL-1 cells (Figure 7B), consistent with the observed decrease in ISL1 and LHX3.

HDACi cause accumulation of histone acetylation, particularly at highly acetylated regions such as SEs. We reasoned that because SE-driven CR TFs were particularly sensitive to HDAC inhibition, looping of SE elements might be disrupted. To test this, we utilized a per-cell spike-in normalization strategy designed to capture absolute quantification of architecture by HiChIP (AQuA-HiChIP) (31) to ensure accurate representation of global acetylation upon HDAC inhibition by panobinostat in the VP-MCC cell lines WaGa and MKL-1. This approach revealed 2 simultaneous phenomena: (a) the loss of SE contacts to both their target gene and adjacent SEs and also (b) a global increase in H3K27ac-mediated contacts seen as a bleeding across the loci in a nonspecific way. These combined changes in chromatin architecture resulted in topological blurring of the SE, as exemplified at the *ISL1* domain (Figure 7C) and other CR TFs (Supplemental Figure 10). Aggregate peak analysis (AQuA-APA) of all adjacent SE pairs showed bloating of H3K27ac-mediated contacts into regions beyond the native SE-SE contacts (Figure 7D). Next, we identified all 3D loops in SEs and regular enhancers (32) and found the anchors of SE loops to be the points of greatest H3K27ac contact loss upon HDAC inhibition, surrounded by areas of increased acetylation-mediated contacts (Figure 7E). The AQuA-HiChIP data, combined with our other results, led us to conclude that VP-MCC CR TFs have a dependence on HDAC1/2/3 without which they are ineffectively transcribed, owing to the topological blurring that occurs with hyperacetylation of SE domains upon HDAC1/2/3 inhibition (Figure 7F). It is possible that H3K27ac HiChIP does not reflect actual topology, as it is enriching for a subset of acetylation-associated 3D conformation. However, we note that in previous work studying HDACi at the MYOD1 SE locus, the topological blurring seen by AQuA-HiChIP of H3K27ac (8) was also seen using 4C-Seq at both the SEs and the gene promoter (27).

## Discussion

We discovered a remarkable cooperative interaction between a host CRC of neuroendocrine TFs and the xenogeneic regulatory sequence of integrated polyomavirus. We provide evidence that VP-MCC is a disease driven by self-reinforced transcriptional networks that have co-opted, and have been co-opted by, an oncogenic polyomavirus, creating a circuit of “lineage-survival oncogenes” (33). Our data strongly suggest that the high levels of MCPyV T antigen oncogenes observed in VP-MCC tumors depend on a host CRC with direct regulation by the CR TFs ISL1 and LHX3. In turn, MCPyV ST and its binding partners MYCL and EP400 co-occupy VP-MCC SEs along with CR TFs and are necessary for VP-MCC transformation and viability (23, 34). MYCL and EP400 both have well-established functions in modifying chromatin and promoting transcription at their targets (35, 36). We have shown that when partnered with MCPyV ST in VP-MCC, these epigenetic effectors are largely directed by neuroendocrine CR TFs. Importantly, the VP-MCC CR TF *INSM1* has been shown to be transcriptionally dependent on the ST-MYCL-EP400 complex in VP-MCC cells (23), further suggesting a positive feedback circuit between neuroendocrine CRC and viral oncogenes.

Our study also characterized SEs in VN-MCC cell lines and contrasted them with VP-MCC cell lines. As previously reported, variant VN-MCC cell lines are more transcriptionally dissimilar to native MCC tumors than classic VP-MCC cell lines (37). This is true even when comparing cell lines with VN-MCC native tumors (Supplemental Figure 2E). Nonetheless, variant VN-MCC lines do represent cells that preferentially grew out from VN-MCC tumors under culture conditions and could be meaningful in the context of treatment-resistant subclones. Further work will be needed to validate our results in the variant VN-MCC cell lines, as they were not a focus of this study and served primarily as a comparator for the VP-MCC cell lines.

Our observation that MCPyV preferentially integrates near-CR TFs and their regulatory elements in VP-MCC may represent another mechanism whereby the polyomavirus and the neuroendocrine lineage cooperate. In models of HPV integration, viral insertion sites disrupt host chromatin interactions to influence local gene expression (38). Positive selection for viral integration events that reinforce neuroendocrine CRC would be consistent with our integration site observations. Alternatively, it is possible that MCPyV integration preferentially occurred at sites of open chromatin, suggesting that the cell of origin for VP-MCC was already expressing neuroendocrine circuitry genes. Further research will be necessary to differentiate these 2 hypotheses. Regardless of how this pattern of integration is established, the dramatic association of integration sites with CRC SEs strongly suggests this is an important feature of VP-MCC carcinogenesis.

Finally, we show a TF addiction (39) in VP-MCC that confers vulnerability to HDAC inhibition that selectively shuts down expression of CR TFs. The 2 mechanisms of oncogenesis in MCC allowed us to compare a mutationally clean, virus-driven, and TF-addicted cancer (VP-MCC) with a more typically mutated oncogene–driven cancer (VN-MCC). In previous work in rhabdomyosarcoma, we have shown how HDACi treatment can halt tumor growth by dramatically reducing the transcriptional output of core TFs under the control of SEs (8). Here, we present a similar regulatory loop and show that HDACi sensitivity is specific to the TF-addicted VP-MCC and has little effect on the mutationally driven VN-MCC.

More broadly, our results for HDACi of SE-driven CR factors shed light on how this class of drugs achieves its remarkable specificity, given its global effects on the epigenome. We propose the concept of overclocked genes, by analogy to overclocked computer processors, where the controls that are used to increase the output of the unit eventually lead to catastrophic failure when pushed too far. A core TF pushed to the limits of its transcriptional output through CR circuitry and SE binding of the ST-MYCL-EP400 complex can similarly collapse when pushed over the edge by the suppression of the deacetylases that had been “cooling” the regulatory loop. The principle of overclocked CR genes extends beyond VP-MCC and lays the foundation for a more general framework to potentially identify opportunities for HDACi treatment in other cancers, subtypes, and, importantly, individual patients in precision oncology.

Finally, the epigenetic pathogenesis of VP-MCC we establish here may be representative for cross-regulation events in other oncovirus-driven cancers. These results highlight the importance of exploring *cis*-regulatory relationships between host and pathogen genomes.

## Methods

### Sex as a biological variable

Sex was not considered as a relevant biological variable. For the human MCC cell lines used in this study, WaGa, MKL-1, and MKL-2 are of male origin, whereas MS-1, MCC13, MCC26, and UISO are of female origin.

### Cell lines and primary tumors

The MCC cell lines used included the VP-MCC lines MKL-1 (40), MKL-2 (41), WaGa (42), and MS-1 (43) and the VN-MCC lines MCC13 (44), MCC26 (44), and UISO (45). Other cell lines used included HEK293T (46), CRL-7250 (47), and NIH-3T3 (48). All cell lines routinely tested negative for mycoplasma, and cell line identities have been ensured by RNA-Seq and short-tandem repeat genotyping. MCC cell lines were grown in RPMI with 10% FBS, 100 U/mLl penicillin and 0.1 mg/mL streptomycin. MCPyV status of the tumors were determined by PCR as previously reported and was confirmed by RNA-Seq (37).

### ChIP assays, ChIP-Seq, and DNase-Seq

ChIP assays were performed using the ChIP-IT High-Sensitivity Kit (Active Motif, catalog 53040). The following antibodies were used: anti-H3K27 (Abcam, catalog ab4729); anti-MATH1/ATOH1 (Thermo Fisher Scientific, catalog PA5-29392); anti-SOX2 (R&D Systems, catalog AF2018); anti-LHX3 (Abcam, catalog ab14555); anti-INSM1 (Santa Cruz Biotechnology, catalog sc-271408); anti-islet1 (Abcam, catalog ab109517); anti-POU4F3 (Abcam, catalog ab58128); and anti-HA tag (Cell Signaling Technology, catalog 2367). ChIP-Seq and DNase-Seq were performed as previously described (47). Further details are provided in the Supplemental Methods.

### SE–gene pair assignment and CRC analysis

Briefly, sequencing reads were mapped to the hg19 reference genome using BWA, and peaks were identified using MACS2, with the exclusion of known blacklisted regions (49). For H3K27ac data, SEs were called using the Rank Ordering of Super-Enhancers 2 (ROSE2) algorithm (https://github.com/BradnerLab/pipeline). Topologically associated domains (TADs) were defined according to boundary calls from Rao et al. (50). Specifically, we obtained boundary locations from 9 cell types analyzed with the Arrowhead tool (Gene Expression Omnibus [GEO] GSE63525) and overlapped them using BEDTools intersect to generate a consensus set of TADs consistently identified across these cell lines. This consensus TAD map is publicly available (https://github.com/GryderArt/AQuA-HiChIP/blob/master/reference_files/TAD_goldstandard.hg19.bed). Assignments of SE-gene pairs were first nominated by requiring the SE and the gene to reside within the same TAD, and by filtering for expression of 10 or more TPM in paired RNA-Seq, as described previously (51). Candidate assignments, primarily TFs (highlighted in Figure 1C), were then validated using enhancer-promoter interactions in H3K27ac HiChIP data from MKL-1 and WaGa cells. All selected SE-gene pairs were supported by called chromatin loops and strong long-range contacts. Loops were identified using the extract_bedpe function in AQuA-Tools, which is publicly available on Github (https://github.com/axiotl/aqua-tools) and described in our online documentation (https://docs.axiotl.com/tools/extract_bedpe/). SEs assigned to CRC TFs were also supported by loops connecting the SE to its target gene, summarized in the APA plot shown in Figure 2D. Associated gene ontology enrichment was performed on SEs using the Genomic Regions Enrichment of Annotations Tool (GREAT) (http://great.stanford.edu/public/html/), an algorithm that performs proximity-based assignment of enhancers to multiple nearby genes prior to statistical evaluation of ontology term enrichments, using the whole human genome as background. Metagene plots and heatmaps were generated using NGSplot (https://github.com/shenlab-sinai/ngsplot).

For CR analysis, we took the SEs from each sample and assigned the nearest TF gene when available within 500 kb, integrating RNA-Seq to exclude TFs expressed at less than 10 TPM from consideration. Then, the motifs for all candidate TFs residing in the valleys of H3K27ac signal were identified and quantified to establish the “in” degree of binding (number of motifs in the SE regulating a given TF) and the “out” degree of binding (the number of other TF SEs that had the motif of a given TF). From this, a network was constructed (https://pypi.org/project/coltron/). TFs that were consistently identified across at least 3 of 4 VP-MCC cell lines and 2 of 3 VN-MCC cell lines were the focus of this study. TFs with a binding degree over 0.5 in at least 2 VP-MCC (VN-MCC) cell lines were identified as VP-MCC (VN-MCC) CR TFs. To identify overlapping genomic locations between different ChIP-Seq experiments, we used bedtools intersect (52). Motif enrichment analysis was performed using HOMER (http://homer.ucsd.edu/homer/motif/). Pipeline code and visualization scripts are available at https://github.com/CBIIT/ChIP_Seq Public ChIP-Seq data sets for ST, EP400 and MAX in MKL-1 cells were downloaded from the GEO database (GEO GSE100183) and analyzed in the same manner as our in-house–generated ChIP-Seq datasets. Raw sequencing data and processed files have been made available through the GEO database (GEO GSE261681).

### DNase1 hypersensitivity assay

A DNase1 hypersensitivity assay was performed to identify regions of open chromatin in VP- or VN-MCC cell lines. Detailed methods are provided in the Supplemental Methods.

### Whole-exome sequencing and analysis of MCPyV integration in MCC

Whole-exome sequencing was performed on 11 of the frozen MCC tumors using a custom array based on the Agilent SureSelect DNA Capture array, to which we added baits that covered the entire MCPyV genome (Agilent Technologies, G9611A plus 5190-0407 oligonucleotide library layout service). The resulting target-captured DNA was subjected to Illumina HiSeq paired-end sequencing. The quality of the resulting genome-aligned sequence was very high: on average, 60 million read pairs were reliably mapped per sample with low (<10%) read duplication rates. The coverage across the captured regions was more than 10 on average across samples. Sequencing reads were aligned to the reference genome, and MCPyV integration sites were manually annotated by identifying human viral split reads and paired-end reads.

### Analyses of the distance between MCPyV integration sites and SEs

To assess the proximity of viral integrations to SEs, the 1,324 SEs present in any of the MCC cell lines were used, and 1,324 random genomic locations were generated as a control using bedtools random. A random subset of SE from a published database of human SEs (21) scaled to cover the same proportion of the total genome was also used as a control. Then, using the coordinates of annotated MCPyV integration sites from 90 patient tumors and cell lines (20, 43, 53–61), the genomic distances between each integration site and the nearest VP-MCC SE, VN-MCC SE, randomly generated genomic locations, or random human SE from the database were calculated. The resulting distances were then plotted in R using ggplot2 (geom_histogram). The median absolute distance to the nearest genomic coordinate and 95% CI were calculated and compared using Student’s *t* test.

### Pooled CRISPR screen using sgRNAs for TFs

MKL-1 cells were seeded into wells of a 24-well plate at a density of 1 × 10^5^ cells in a volume of 700 μL. A solution of 0.7 μL of 8 mg/mL stock polybrene and 140 μL concentrated Cas9-puro virus (https://www.addgene.org/108100/) was added to each well, and the cells were incubated for 18 hours, after which the media were changed to RPMI 1040, 100 U/mL penicillin plus 0.1 mg/mL streptomycin, and 2 μg/mL puromycin. Cells were split 1:2 after 2 days and then passaged routinely to maintain a population of Cas9-puro–infected cells. Western blotting was performed to confirm Cas9 expression (Abcam, Ab204448 antibody). Cas9-expressing MKL-1 cells were transduced with TF sgRNAs for dropout CRISPR screening as described previously (62). MKL-1 cells were plated at a concentration of 20.7 million cells per flask in 3 flasks. Two days after transduction, cells were counted, and 1 million were fixed in 4% PFA for 10 minutes, washed in 1× PBS, and resuspended in 1× PBS for flow cytometric analysis using GFP to verify sgRNA transduction. Forty million cells were pelleted, and 25 million MKL-1 cells were replated. Transduced cells were then consistently passaged, and at each passage, 40 million cells were frozen and 20 million replated. DNA was extracted from pellets using the Qiagen DNeasy Blood and Tissue Kit (Qiagen, catalog 69506), and PCR was performed to amplify integrated sgRNA content (LRG_F2: 5′-TCTTGTGGAAAGGACGAAACACCG-3′; LRG_R2: 5′-TCTACTATTCTTTCCCCTGCACTGT-3′; 5 μL template DNA at 100 ng/μL, 2 μL of 5 μM each LRG_F2/R2, 25 μL PCR High-Fidelity Master Mix, 18 μL water) at PCR conditions of 98°C for 2 minutes, (98°C for 8 seconds, 65°C for 12 seconds, 72°C for 10 seconds) for 28 cycles, then 72°C for 5 minutes. Twenty-four such reactions per time point were performed in parallel and pooled before library preparation using the TruSeq ChIP Library Preparation Kit (Illumina, Set A, IP-202-1012), followed by sequencing (Illumina NextSeq). The abundance of each sgRNA was tabulated for each time point using MAGeCK (63) and visualized in R (https://github.com/GryderArt/CRISPRtoolkit/).

### CRISPRi of SEs

To validate the functional requirement for called SE loops in 2 CR TFs, sgRNAs were synthesized, annealed, and cloned into a pLV hU6-sgRNA hUbC-dCas9-KRAB-T2a-GFP (Addgene plasmid 71237) vector for CRISPRi experiments. MKL-1 cells were nucleofected using the SF Cell Line 4D-Nucleofector X Kit L (24 RCT, catalog V4XC-2024) and the nucleofector program CM137 with dCas9-KRAB plasmid containing a single negative control sgRNA or an sgRNA targeting the specified enhancer element. Reverse transcription quantitative PCR (RT-qPCR) for target gene expression was performed 48 hours after nucleofection.

The targeted enhancer locations are: INSM1_E1: chr20, 20196393-20197546; INSM1_E2: chr20, 20420996-20421817; INSM1_E3: chr20, 20528998-20529950; POU4F3_E1: chr 5, 145710435-145711774; and POU4F3_E2: chr5, 145788400-145789409.

The 5′ to 3′ sequences for the sgRNA oligonucleotides used for cloning are: INSM1_e1FWD: CACCGAATGCTACACAGTTATGGG; INSM1_e1REV: AAACCCCATAACTGTGTAGCATTC; INSM1_e2FWD: CACCGTAGCCATTCTAGGGTTAACG; INSM1_e2REV: AAACCGTTAACCCTAGAATGGCTAc; INSM1_e3FWD: CACCGAGCCTAGGGCATTAATCAG; INSM1_e3REV: AAACCTGATTAATGCCCTAGGCTC; POU4F3_e1FWD: CACCGTGGTAGGCATTCCTTACGA; POU4F3_e1REV: AAACTCGTAAGGAATGCCTACCAC; POU4F3_e2FWD: CACCGGTCGTCATGCGGTAGACAG; and POU4F3_e2REV: AAACCTGTCTACCGCATGACGACC.

### Dual luciferase assays

The MCPyV noncoding control region was cloned from MKL-1 genomic DNA. Dual luciferase reporter assays were conducted in HEK293T cells to assess the activity of the *ISL1/LHX3* homeodomain motif within MCPyV sequence. Detailed methods are provided in Supplemental Methods.

### AQuA-HiChIP

Absolute Quantification of Architecture HiChIP (AQuA-HiChIP) was performed on the MCC lines MKL-1 and WaGa, which were treated with DMSO or 300 nM panobinostat for 6 hours. A detailed step-by-step protocol has been published by our group (31, 64). Briefly, cells were resuspended and counted in triplicate, and identical numbers of cells in all conditions (8 million each) were fixed with 1% formaldehyde, quenched with glycine, pelleted at 4°C, and washed with cold PBS. Murine NIH3T3 fixed and frozen cells (2.5 million) were resuspended in PBS and added to each human cell line condition. Cells were then permeabilized with 0.5% SDS (in TE pH 7.4) for 10 minutes at 62°C, quenched with Triton X-100 (10% in TE pH 7.4), and digested with Dpnii (400 units, New England Biolabs [NEB]) for 2 hours at 37°C. Samples were heat inactivated (20 minutes at 62°C), followed by addition of biotin–14-dATP (Thermo Fisher Scientific, 19524-016) and DNA PolI Large Klenow fragment (NEB, M0210), and then heated to 37°C for 1 hour, followed by addition of T4 DNA ligase (4 hours at 25°C) to incorporate biotin into the Dpnii cleaved overhangs and ligate the proximal chromatin conformations. Nuclei were pelleted and resuspended in 0.2% SDS in TE and sonicated in a cooled water bath (Diagenode Bioruptor, 30 seconds on/30 seconds off, medium setting) until a fragment size enriched in the range of 300–2,000 bp was achieved. Buffer was adjusted to RIPA, and immunoprecipitation was performed using anti-H3K27ac antibody (Active Motif, 39133) overnight, with rotation at 4°C. Protein A Dynabeads were added for the final 2 hours of rotation at 4°C, followed by washing (the same as ChIP-Seq), elution with SDS, and proteinase K treatment for 30 minutes at 55°C. Then, adjusting to RIPA, the samples were heated at 65°C overnight to reverse crosslinks. DNA was purified with the Zymo ChIP-clean and concentrator kit, eluted in 12 μL water, and incubated with Streptavidin Dynabeads M-280 (Thermo Fisher Scientific, 11205D). Libraries were prepared on-bead, with end-repair, A-tailing, adapter ligation, and library amplification. DNA libraries were purified with AMPure beads, and multiplexed libraries were sequenced to a depth of 200 million paired-end reads each (Illumina NextSeq High Output, 150 cycles). Duplicate reads, self-ligated products, and invalid contacts were removed, and the remaining mapped contacts were used to generate genome-wide contact matrices. Mouse (mm10) and human (hg19) contacts were counted for each sample and used to calculate a global spike-in correction AQuA-factor (as performed previously, with code available at GitHub at https://github.com/GryderArt/AQuA-HiChIP). Matrix and APA plots were AQuA normalized using condition-specific AQuA factors for each cell line. We used the HiC-Pro^1^ v 3.0.0 to align paired-end reads independently to the hg19 reference genome, using bowtie parameters global (–very-sensitive -L 30 –score-min L,-0.6,-0.2 –end-to-end –reorder) and local (–very-sensitive -L 20 –score-min L,-0.6,-0.2 –end-to-end –reorder). Pair-end mates went through a robust quality control process that rescued chimeric fragments and removed singleton and multiple-mapping reads. Reads were then assigned to a restriction fragment that allowed for filtering of invalid ligation products. The obtained allValidPairs files were then used to call loops. We used cLoops^2^ with the suggested parameters (-eps 2500, 5000, 7500, 10000 -minPts 10, 15, 20) to detect significant loops from around 50 million Pair End Tags (PETs).

### Small-molecule compounds

The HDACi panobinostat was supplied by the NCI’s Developmental Therapeutics Program (NIH). For in vitro experiments, panobinostat was dissolved in DMSO to a concentration of 10 mM and then diluted to a final volume of less than 0.01% DMSO for all cell culture experiments.

### HDACi viability response

We used VP-MCC cell lines (WaGa, MKL-1, and MKL-2), VN-MCC cell lines (MCC13, MCC26, and UISO), and control cell lines (HEK293T, CRL-7250, and NIH-3T3) as described above. RPMI 1640, DMEM, PBS, FBS, penicillin-streptomycin solution, and trypsin were used for cell culturing (all from Thermo Fisher Scientific). Accutase (MilliporeSigma) was used for cell dissociation.

Sixteen HDACi compounds were tested (see names in Supplemental Figure 7). Cell lines were treated with 11 doses per compound, measuring cell viability after 72 hours. Cell lines were dissociated with trypsin or accutase (for MKL-1 and MKL-2), filtered through a 40 μm cell strainer, and plated into multi-well plates. The starting density ranged from 50 cells/μL (HEK293T), 80 cells/μL (NIH-3T3), 100 cells/μL (MKL-1, MKL-2, MCC13, MCC26, CRL7250, and UISO), to 250 cells/μL (WaGa) in a final volume of 5 μL media (MCC cells: RPMI 1640; control cells: DMEM) supplemented with 10% FBS and 1× penicillin/streptomycin. Compound (23 nL) in DMSO was then transferred to the assay plates (65). After 72 hours of incubation at 37°C and 5% CO_2_, 2.5 μL CellTiter-Glo (Promega) was dispensed into each well. Plates were incubated at room temperature for 10 minutes and transferred to a ViewLux (PerkinElmer), and the luminescence was recorded using an exposure time of 2 seconds. Relative luminescence units (RLU) were normalized to in-plate controls (no cells as a positive control, DMSO as a negative control), and the normalized data were processed and analyzed using NCATS in-house software (66). In-plate controls were used for the calculation of the Z′-factor index for each assay. The area under the dose-response curve (AUC) was calculated for each compound using the trapezoidal formula. AUCs for duplicate compounds were averaged. AUCs below 0 were set to 0.

The effect of panobinostat on MKL-1 cell viability and apoptosis was assessed using the CellTiter-Glo Luminescent Cell Viability Assay and the Caspase-Glo 3/7 Assay System, respectively (Promega). Detailed methods are provided in the Supplemental Methods.

### siRNA nucleofection, RNA extraction, and RT-qPCR

Nucleofection of VP-MCC cell lines with siRNA constructs was done using the Lonza SG Cell Line 4D-Nucleofector X Kit L (24 reactions) (catalog V4XC-3024) and the nucleofector program CM138 for WaGa cells, and the SF Cell Line 4D-Nucleofector X Kit L (24 RCT) (catalog V4XC-2024) with the nucleofector program CM137 for MKL-1 cells, according to the manufacturer’s instructions. siRNAs (Dharmacon) included ON-TARGETplus Cyclophillin B Control Pool – Human (catalog D-001820-10-05); ON-TARGETplus Nontargeting Pool (catalog D-001810-10-05); SMARTpool: ON-TARGETplus ISL1 siRNA (catalog L-011707-00-0005); and SMARTpool: ON-TARGETplus LHX3 siRNA (catalog L-013588-00-0005). siRNAs were nucleofected at a concentration of 1 μM/reaction using 1 million cells per nucleofection. RNA was extracted 48 hours after nucleofection, and RT-qPCR was performed to quantify the expression of ISL1, LHX3, and the MCPyV LT and ST. Further details are provided in the Supplemental Methods.

### Nucleofection of HA-tagged ST, immunostaining, and imaging

Nucleofection of the VP-MCC MKL-1 cell line with 1 μg each of HA-tagged ST or empty vector control was done using the SF Cell Line 4D-Nucleofector X Kit L (24 RCT) (catalog V4XC-2024) and the CM138 program. Forty-eight hours after nucleofection, MKL-1 cells were spun down onto coverslips (Thermo Fisher Scientific, 3305) via CytoSpin. Cells on coverslips were stained and imaged using the Instant Structured Illumination Microscope (iSIM) system. The following primary antibodies were used: anti-islet1 (Abcam, ab109517, 1:100); anti-LHX3 (Abcam, ab14555, 1:100); anti-SOX2 (Stemgent, 09-0024, 1:100); anti-POU4F3 (Novus Biologicals, NBP1-88349, 1:100); anti-p400 (Bethyl Laboratories, A300-541A, 1:100); anti–HP1-α (Abcam, ab109028, 1:100); and anti–HA tag (Cell Signaling Technology, catalog 2367s,1:100). Further details are provided in the Supplemental Methods.

### Animal studies

Mouse xenograft studies were performed at the Frederick National Laboratory. Female, athymic nu/nu mice, 4–6 weeks of age, were obtained from Charles River Laboratories and housed at the Frederick National Laboratory for Cancer Research. Mice were s.c. injected with 10 million MKL-1 cells (25 gauge needle) or 5 million MCC13 cells (23 gauge needle) in 50% Matrigel (Corning) in PBS for a total volume of 100 μL (MKL-1) or 300 μL (MCC13). When the average tumor volume reached 100 mm^3^, the animals were assigned to treatment groups using the StudyLog software randomization tool and were treated with either 10 mg/kg body weight panobinostat or 5% DMSO in RPMI vehicle control by i.p. injection. Treatments were administered daily, Monday through Friday, during the first week followed by Monday, Wednesday, Friday dosing during weeks 2–4. Tumor size and body weight were measured on average 2 times or 3 times per week, respectively. After 4 weeks of treatment, tumor size and body weight were monitored twice a week for an additional 4 weeks. Animals were euthanized when they reached a study endpoint of body weight loss exceeding 20%, a maximum tumor diameter exceeding 20 mm, or the end of the study.

### RNA-Seq of panobinostat-treated MCC cells

One million MKL-1 cells or MCC26 cells were treated with 0.3 μM panobinostat or control DMSO for 1 or 6 hours, after which RNA was harvested for RNA-Seq. RNA extraction was performed as described in the Supplemental Methods. Sequencing was performed on the Illumina HiSeq platform, generating approximately 30 million paired-end reads per sample. Reads were mapped using STAR, gene-level TPM were counted with RSEM, and GSEA analysis was performed comparing DMSO with panobinostat at 1 and 6 hours. The pipeline scripts for RNA analysis and visualization are available in GitHub (https://github.com/GryderArt/VisualizeRNASeq). Identical RNA-Seq protocols and analyses for expanded HDAC isoform selective inhibitors were performed with panobinostat (500 nM), mocetinostat (5 μM), and dacinostat (1 μM) for 6 hours, with DMSO controls, in MKL-1, MKL-2 and WaGa cells.

### Western blot analysis

Western blotting was performed to assess cleavage of PARP and caspase 3 in panobinostat-treated MKL-1 cells. The following primary antibodies were used: PARP (9532, 1:1,000, Cell Signaling Technology); cleaved caspase 3 (9664, 1:1,000, Cell Signaling Technology); pro–caspase 3 (sc-56053, 1:200, Santa Cruz Biotechnology); and β-actin (sc-47778, 1:200, Santa Cruz Biotechnology). Detailed methods are provided in the Supplemental Methods.

### Statistics

Statistical analyses were conducted using GraphPad Prism (GraphPad Software). Data in the figures are presented as the mean ± SEM unless otherwise noted. A *P* value of less than 0.05 was considered statistically significant. *P* values for comparison between 2 groups were calculated using 2-tailed Student’s *t* test. For comparison between multiple groups, *P* values were calculated by 1-way ANOVA.

### Study approval

#### Patient samples.

Fresh-frozen MCC tumor specimens were collected at Memorial Sloan Kettering Cancer Center under IRB protocol 00-144 A. All patients provided written informed consent for the use of their samples. Analysis of samples was conducted under NCI protocol 13CN024 without obtaining further consent, as patients had provided prior consent, and the samples were analyzed anonymously.

#### Animal study.

The animal research performed was approved by the IACUCs of the NCI and the NIAMS, NIH. Animal facilities were accredited by AAALAC International and followed the Public Health Service Policy for the Care and Use of Laboratory Animals. Animal care was provided in accordance with institutional guidelines and the procedures outlined in the *Guide for Care and Use of Laboratory Animals* (National Academies Press 2011).

### Data availability

ChIP-Seq, DNAse-Seq, AQuA-HiChIP data are available through the GEO database (accession GSE261681). RNA-Seq data from MCC tumors are available in the Database of Genotypes and Phenotypes (dbGaP) (accession phs003411.v1.p1; https://www.ncbi.nlm.nih.gov/projects/gap/cgi-bin/study.cgi?study_id=phs003411.v1.p1). Code used in this study is available on GitHub (https://github.com/GryderArt/AQuA-HiChIP) as described in Methods.

## Author contributions

BEG, JK, and IB conceived the study. BEG wrote the manuscript. All authors edited the manuscript. AC, DM, TG, LM, KAG, LC, NTH, KD, YS, RC, and BEG performed wet lab experiments. DM performed all luciferase experiments, constructed all related plasmids, and performed all CRISPR-KRAB experiments. AC and LM performed RT-qPCR experiments and all superresolution imaging experiments. KJB diagnosed and acquired MCC tumor samples. CRV designed and provided the CRISPR guide library. VG, KD, AK, AM, JS, JP, MC, RS, and BEG performed bioinformatics analysis. DJU, TG, MS, and MDH designed and conducted the HDACi viability screen. SV, DR, and TA performed data curation. BEG, JK, and IB supervised the project.

## Funding support

This work is the result of NIH funding, in whole or in part, and is subject to the NIH Public Access Policy. Through acceptance of this federal funding, the NIH has been given a right to make the work publicly available in PubMed Central.

- National Institute of Arthritis and Musculoskeletal and Skin Diseases.
- National Cancer Institute (NCI).
- National Center for Advancing Translational Sciences Intramural Research Programs of the NIH.
- Department of Defense Civil Money Penalty Reinvestment Program Convergent Science Virtual Cancer Center (W81XWH-21-1-0298).
- NCI (1R01CA291963-01) to BEG.

## Supplementary Material

Supplemental data

Unedited blot and gel images

Supplemental table 1

Supplemental table 2

Supporting data values

## Version 1 12/15/2025

Electronic publication

## Version 2 01/12/2026

updated accession information
