# Supplementary material for: Interlocking host and viral cis-regulatory networks drive Merkel cell carcinoma
Source: J Clin Invest. 2025 Dec 15;135(24):e188924. doi: 10.1172/JCI188924 (PMC12700541; doi:10.1172/JCI188924)
Supplement: Supplemental data [file jci-135-188924-s008.pdf]

Supplemental Material for

Interlocking host and viral cis-regulatory networks drive Merkel cell carcinoma

This PDF file includes:

Supplemental Methods
Supplemental Figures 1–10
Legends for Supplemental Tables 1 and 2

Supplemental Methods

ChIP assays

Cells were plated at a concentration of 1.5 x 10^7 in 10 cm tissue culture plates with 15 mL of RPMI + 10% Fetal Bovine Serum + Pen/strep, and grown to 70-80% confluency. For ST ChIP-seq in HEK293 cells, cells were transfected with HA-small T plasmid 24 hours prior to ChIP. Cells were fixed and fixation stopped according to manufacturer's protocol. VN-MCC adherent cells were scraped, transferred to a 50 ml tube, while VP-MCC suspension cells were transferred directly to a 50 ml tube. Cells were counted, pelleted for 3 minutes at 1250g at 4°C, and washed twice in PBS wash buffer according to manufacturer's protocol. Supernatant was removed and cell pellets were frozen at -80°C for future use. Cell pellets were thawed and resuspended in Chromatin Prep Buffer with PIC and PMSF according to manufacturer's protocols, dounce homogenized on ice for 50 strokes and allowed to sit for bubbles to settle out. After homogenization, cells were sonicated at 30% amplitude for 30 seconds "on" and 30 seconds "off" in each of 20 cycles (using Active Motif's EpiShear platform), while mounted on a tube cooler. Samples were divided into 2 tubes of 25 µL (input) and 3 tubes of 200 µL and stored at -80°C. 25 µL samples were used for analysis of shearing efficiency of chromatin, as well as for quantification, which was done according to manufacturer's protocol, and agarose gel showed successful chromatin shearing. Chromatin Immunoprecipitation was also done according to manufacturer's protocol with minor adjustments. Briefly, 200 µL of sonicated chromatin from VP-MCC and VN-MCC cells was thawed on ice. 5 µL of blocker and 10 µL of H3K27 antibody (Abcam, cat. no. ab4729) or 15 µL of anti-MATH1/ATOH1 (Thermo Fisher, cat. no. PA5-29392), anti-SOX2 (R&D Systems, cat. no. AF2018), anti-LHX3 (Abcam, cat. no. ab14555), anti-INSM1 (Santa Cruz, cat. no. sc-271408), anti-Islet1 (Abcam, cat. no. ab109517), anti-POU4F3 (Abcam, cat. no. ab58128), or anti-HA tag (Cell Signaling, cat. no. 2367) was used per reaction. Reversal of crosslinking and ChIP DNA Purification was also done according to manufacturer's directions. DNA was stored at -80°C for later analysis.

DNase1 hypersensitivity assay

Buffers for the DNase1 assay were as follows: RSB buffer (10mM Tris, 10 mM NaCl, 3mM $MgCl_2$) on ice, 2X RSB lysis buffer (9.95 mL RSB buffer, 50 µL 20% Triton X-100) on ice, 1X RSB lysis buffer (1 mL RSB buffer, 1 mL 2x RSB lysis buffer) on ice and Stop buffer (9.5 mL H2O, 100 µL 1M TrisHCL pH 7.4, 20 µL 5M NaCl, 200 µL 0.5M EDTA) at room temperature. 1 million cells from each VP-MCC (MKL-1, MKL-2, MS-1, and WaGa)

and VN-MCC (MCC13, MCC26, and UISO) cell lines were resuspended in 1X PBS and placed on ice. 100 µL of each cell suspension (100K cells) was aliquoted into 8 PCR tubes and pelleted at 1230 RPM x 7 minutes at 4°C. 90 µL of supernatant was removed, cells were washed 2x in 200 µL RSB buffer, and cells were resuspended in 190 µL RSB buffer. Cells were divided into tubes for lysis and DNase 1 treatment as follows: 20 µL (10K) cells/tube + 20 µL 2X RSB lysis buffer. Tubes were vortexed, lysed at room temp x 1 minute, and placed at 4°C. Cells were lysed in 1X RSB lysis at room temperature for 1 minute, and placed at 4°C. 15 µL/mL 10% SDS and 1.25 U/µL Proteinase K (Roche, cat. no. 03-115-828-001) were added to stop buffer. 10 U/µL DNase1 (Roche, cat. no. 04-716-728-001) was diluted to 60x, 90x, or 120x in 1xRSB lysis buffer on ice. 10 µL of 60x, 90x, or 120x DNase1 was added to tubes of lysed cells, which were vortexed for 2 seconds, incubated at 37 °C x 5 minutes, after which 50 µL of Stop buffer was added to halt the reaction. Samples were frozen at -80 °C until ready for DNA purification.

Dual luciferase assays

The MCPyV noncoding control region was cloned from MKL-1 genomic DNA. The selected region was amplified using Q5 high fidelity polymerase (New England Biolabs, cat. no. M0492S) using the following primers (5' to 3'): Forward TACCTGAGCTCGCTAGCTGTCCTCCTCCCTTTGTAAGAGA; Reverse TTTGGCATCTTCCATGGTGTCTATATGCAGAAGGAGTTTGCAG. The 426 bp product was then cloned into the NheI and NcoI sites of the pGL4.10 luciferase reporter plasmid (Promega, cat. no. E6651). Site directed mutagenesis was performed using the GeneArt Site-Directed Mutagenesis System (Invitrogen, cat. no. A13282) according to the manufacturer's instructions using the following primers (5' to 3'): Forward CTGAGGCTTAAGAGGGATATCTAGCAAAAAAGGCAG; Reverse CTGCCTTTTTTGCTAGATATCCCTCTTAAGCCTCAG. HEK293T cells were co-transfected with a pGL4.10-Firefly reporter construct, an internal control pGL4.75_cmv-Renilla reporter (Promega, cat. no. A13282) using Viafect transfection reagent (Promega, cat. no. E4982), and human cDNA expression plasmids for ISL1 or LHX3 (GeneScript, cat. no. OHu01644 or OHu19223, respectively). Transfections were performed in triplicate. Relative luciferase activity was quantified using the Dual Luciferase Assay Reporter System (Promega, cat. no. E1910) according to the manufacturers' instructions.

RNA extraction and RT-qPCR

RNA was extracted at 48 hours after nucleofection using the RNeasy minikit (Qiagen, cat. no. 74134) according to manufacturer's instructions. Qiashredder columns (Qiagen, cat. no. 79654) were used for cell disruption and homogenization according to manufacturer's instructions. DNA was digested following the optional DNase digestion protocol (RNeasy Mini kit protocol, part 2) as described, using the RNase-Free DNase set (Qiagen, cat. no. 79254) according to manufacturer's instructions. RNA quality and concentration were analyzed using the Agilent Bioanalyzer with the RNA 6000 Nano kit (Agilent, cat. no. 5067-1511) according to manufacturer's instructions, and RNA was stored at -80°C for later use.

cDNA was synthesized with the Superscript III First-Strand Synthesis System (ThermoFisher Scientific, cat. no. 18080051) according to manufacturer's instructions. The first set of cDNA was synthesized using oligo(dT)20 primers and 100 ng of RNA, while subsequent cDNA synthesis was done using random hexamers and 200 ng of RNA. cDNA was stored at -80°C.

QuantiTect SYBR Green PCR Kit (Qiagen, cat. no. 204143) was used for qPCR of LHX3 and ISL1 according to manufacturer's instructions. 1 µL of template cDNA was used in each reaction. QuantiTect Primer Assays were as follows: LHX3 (Qiagen Hs_LXH3_1_SG QuantiTect Primer Assay, cat. no. QT00026579), ISL1 (Qiagen Hs_ISL1_1_SG QuantiTect Primer Assay, cat. no. QT000002940), and B2-Microblobulin (Qiagen Hs_B2M_1_SG QuantiTect Primer Assay product # 249900, cat. no. QT00088935). Reactions were run in an Applied Biosystems StepOne Plus Real Time System for 40 cycles as follows: Denature for 15 min x 95°C, 40 cycles of 15 sec x 94°C, 30 sec x 55°C, 30 sec x 72°C, followed by a melt curve.

qPCR for T-antigen was performed with power SYBR Green PCR master mix (Thermo Fisher Scientific) and analyzed on StepOnePlus real-timePCR system (Thermo Fisher Scientific). The relative T-antigen and CRTF mRNA levels were analyzed by normalizing the threshold cycle (Ct) value to that of internal loading control, β2-microglobulin (*B2M,* n=3). Primers for T-antigen quantification are: small T forward 5'-GTTGTCTCGCCAGCATTGTAGTC-3'; small T reverse 5'-CCACCAGTCAAAACTTTCCCAAG-3'; large T forward 5'-TTCCTCTGGGTATGGGTCCTTC-3'; large T reverse 5'-TTGGTGGTCTCCTCTCTGCTACTG-3'; B2M forward 5'-CCACTGAAAAAGATGAGTATGCCT-3'; B2M reverse 5'-CCAATCCAAATGCGGCATCTTCA-3'.

Immunostaining and imaging

Cells on coverslips were fixed in freshly prepared 4%PFA for 10 minutes at room temperature and blocked with 10% serum in PBS with 0.1% Triton X-100 (PBT) for 30 minutes and then incubated in primary antibody (diluted in PBT with 5% serum) overnight at 4°C. Alexa Fluor-conjugated secondary antibodies (1:1000, Thermo Fisher Scientific) were then applied. Control staining where the primary antibody was omitted were used to confirm the specificity of experimental staining. Cells on coverslips were mounted onto slides using ProLong™ Glass Antifade Mountant (Thermo Fisher Scientific, cat. no. P36980). Instant Structured Illumination Microscope (iSIM) imaging was conducted on VT-iSIM (VisiTech International) module integrated with a Nikon Ti base. Cells were imaged using a 100× 1.49 numerical aperture (NA) oil objective and sCMOS camera (ORCA-Fusion BT; Hamamatsu). Green fluorescence was collected using a 525/50 emission filter (Semrock) and red fluorescence was collected using a 605/52 filter (Semrock). The camera, lasers and microscope are controlled by Nikon NIS-Elements Imaging software (Version 5.30). Images were deconvolved using Richardson–Lucy algorithm through customer written MATLAB (MathWorks) script.

Western blot

Protein concentration was assessed using the Bradford assay (Bio-Rad Protein Assay Dye Reagent Concentrate, Bio-Rad). The protein samples were prepared in 5X Western-Ready Protein Sample Loading Buffer (BioLegend) containing 4% β-mercaptoethanol, separated on 4-12% NuPAGE Bis-Tris protein gels (Thermo Fisher Scientific), and transferred to a PVDF membrane (Thermo Fisher Scientific). The PVDF membrane was blocked for 10 minutes with EveryBlot blocking buffer (Bio-Rad) before a 1-hour incubation with primary antibodies against PARP (1:1000, Cell Signaling, cat. no. 9532), cleaved caspase-3 (1:1000, Cell Signaling, cat. no. 9664), procaspase-3 (1:200, Santa Cruz Biotechnology, cat. no. sc-56053), or β-actin (1:200, Santa Cruz Biotechnology, cat. no. sc-47778). After primary antibody incubation, membranes were washed with TBS (or TBST for cleaved caspase-3 and procaspase-3) (Thermo Fisher Scientific).

For PARP and β-actin, membranes were incubated for 1 hour with Alexa Fluor 488 goat anti-rabbit IgG (H+L) cross-adsorbed secondary antibody (Thermo Fisher Scientific, cat. no. A-11008) and Alexa Fluor 546 goat anti-mouse IgG (H+L) cross-adsorbed secondary antibody (Thermo Fisher Scientific, cat. no. A-11003), respectively. For cleaved caspase-3 and procaspase-3, membranes were incubated for 1 hour with HRP-conjugated

goat anti-rabbit secondary antibody (1:1000, Cell Signaling, cat. no. 7074) and HRP-conjugated goat anti-mouse secondary antibody (1:1000, Cell Signaling, cat. no. 7076), respectively.

After secondary antibody incubation, membranes were washed with TBS or TBST. Finally, for cleaved caspase-3 and procaspase-3, the membranes were treated with HRP substrate (Pierce ECL Western Blotting Substrate, Thermo Fisher Scientific) for 5 minutes. All membranes were visualized using the iBright FL1500 Imaging System (Thermo Fisher Scientific).

CellTiter-Glo and Caspase-Glo Assays

MKL-1 cells were plated at a concentration of 2.5 × 105 cells/mL in Falcon 96-well white-walled clear flat-bottom plates (Corning Inc.) in quadruplicate and treated with 300 nM panobinostat for 6 and 24 hours. Cell viability and apoptosis were assessed using the CellTiter-Glo Luminescent Cell Viability Assay and the Caspase-Glo 3/7 Assay System (Promega), respectively. At the time of luminescence measurement, plates were equilibrated to room temperature for 30 min, followed by the addition of 100 µL of either CellTiter-Glo reagent or Caspase-Glo 3/7 reagent per well. Luminescence was measured using a Victor X3 Multilabel Reader (Beckman Coulter) and values were normalized to DMSO controls. Data represent the average of 4 replicates.

Supplemental Figures 1–10

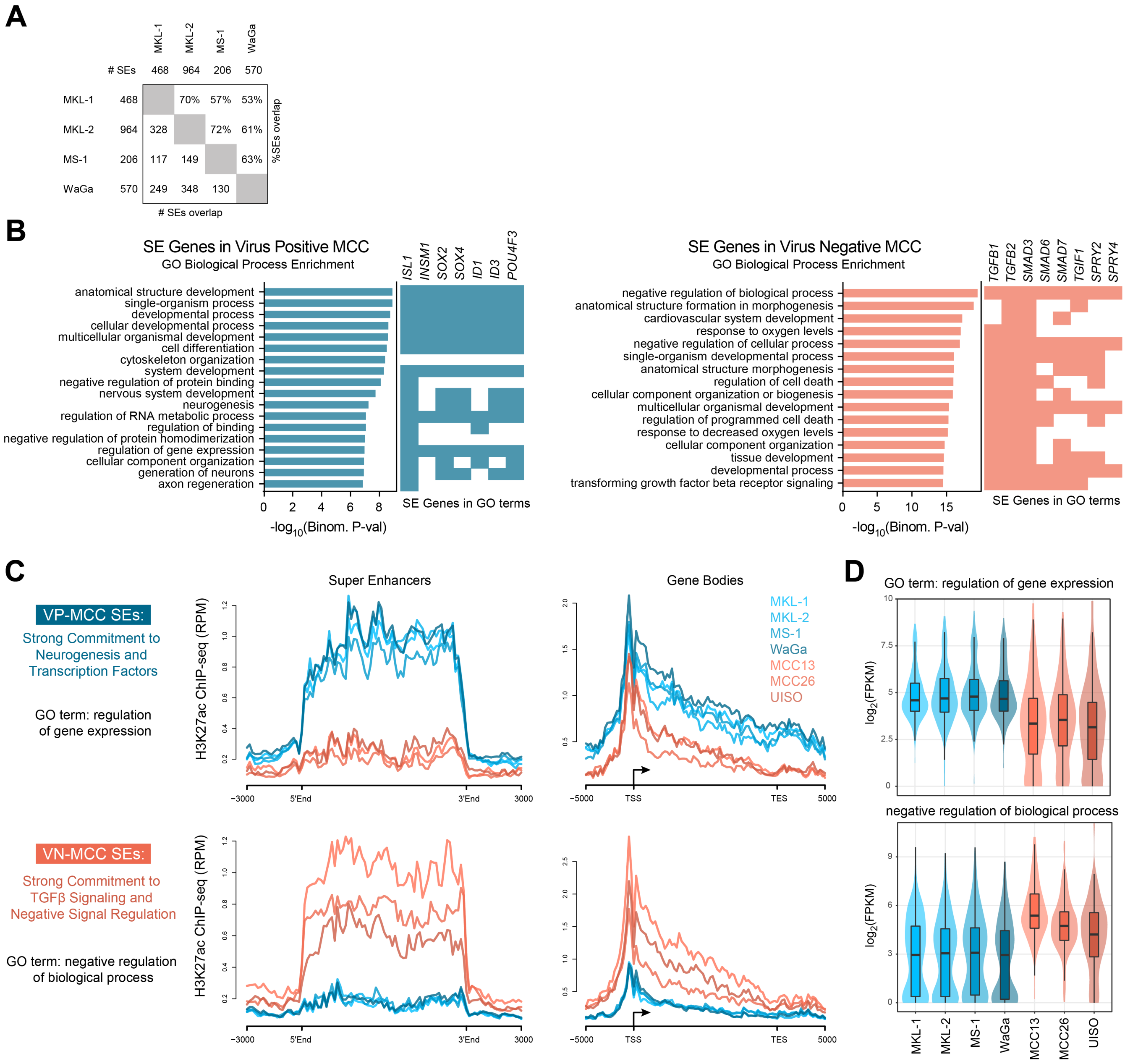

Supplemental Figure 1. Divergent SE landscapes of MCC reveal subtype specific commitments

A. The number of SEs identified in each VP-MCC cell line, and a matrix of their total number of overlapping SEs (bottom left) and the percentage of overlapping SEs (top right). The percent overlapping SE among the VP-MCC cell lines is notably larger than the 8.8% overlapping aggregate SEs between VP-MCC and VN-MCC.

B. Gene ontology (GO) for Biological Processes enriched in SE associated genes. The presence or absence of select genes included in each gene set is indicated by colored boxes.

C. H3K27ac ChIP-seq metaplots for SEs and their associated gene bodies present in characteristic gene ontology sets for VP-MCC (top) and VN-MCC (bottom). VP-MCC SEs drive a strong transcriptional commitment to Neurogenesis pathway genes and Transcription Factors (TFs) while VN-MCC SEs drive a strong transcriptional commitment to TGFβ Signaling and Negative Signal Regulation genes. RPM; reads per million.

D. Gene expression within top Gene Ontology (GO) terms are enriched in a viral-status selective manner. FPKM; fragments per kilobase per million mapped reads.

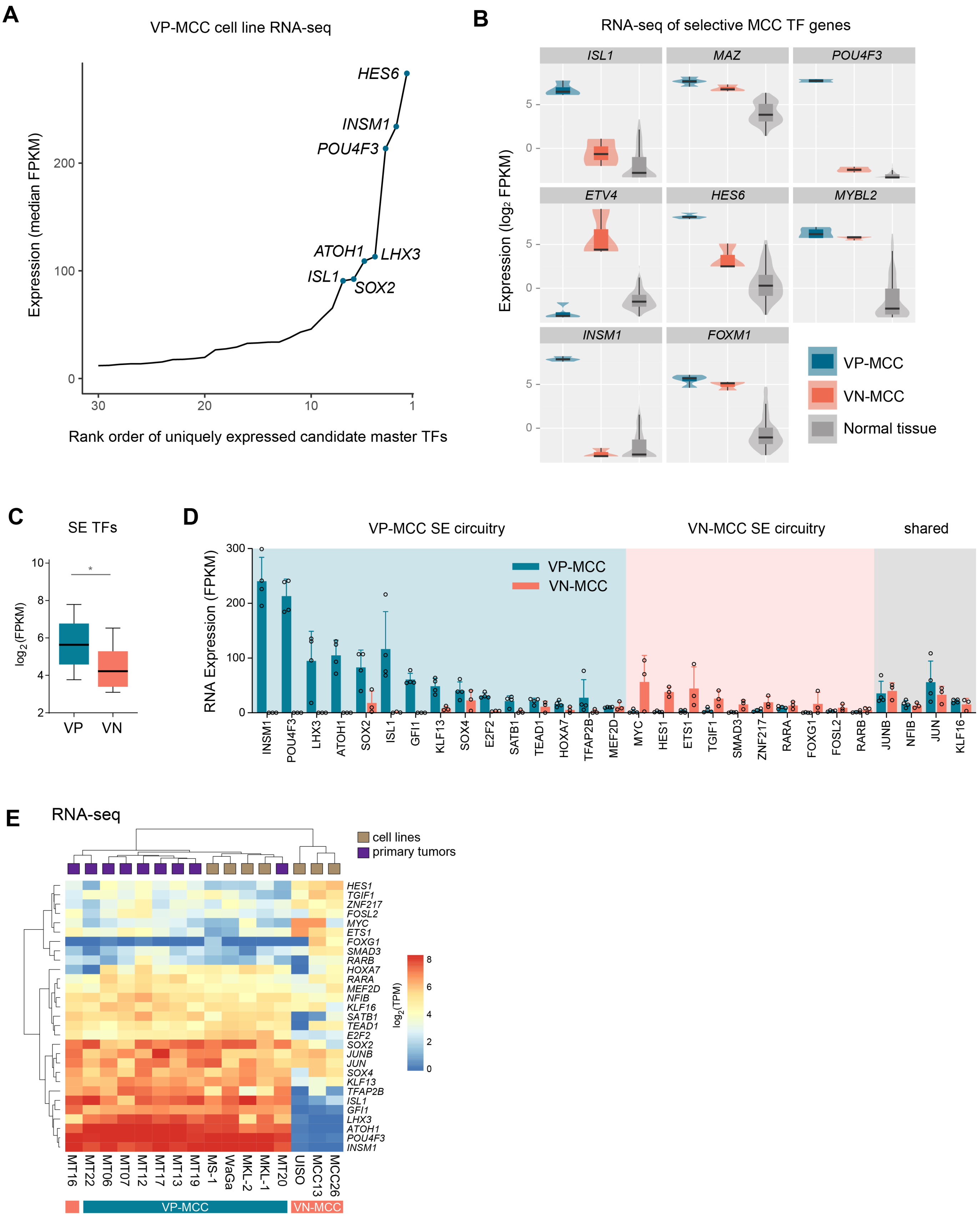

Supplemental Figure 2. RNA-seq reveals high quantity and abundance of TFs in VP-MCC

A. RNA-seq analysis of 30 transcription factor genes selectively expressed in VP-MCC cell lines (log2 fold change greater than 3, which is a greater than 8-times greater abundance in VP- over VN-MCC cell lines, by median FPKM), ranked by median FPKM expression in VP-MCC cell lines.
B. Violin with overlaid boxplots revealing distinct distributions of expression for select TFs over-expressed in either MCC subtype (n = 4 VP-MCC, n = 3 VN-MCC), as compared to normal human tissue RNA-seq (n = 222 diverse normal tissues).
C. SE-associated TFs in VP-MCC are expressed at higher levels than SE-associated TFs in VN-MCC. Box plots show median $\log_2$(FPKM) expression of all TFs among all cell lines within each MCC subtype. * $P < 0.01$ by students t-test.
D. Mean expression of individual core regulatory TFs for VP-MCC (n=4 independent samples) and VN-MCC (n = 3 independent samples) subtypes.
E. Clustered heatmap of CR TF RNA-seq expression from MCC cell lines and tumors. TPM, Transcripts Per Million.

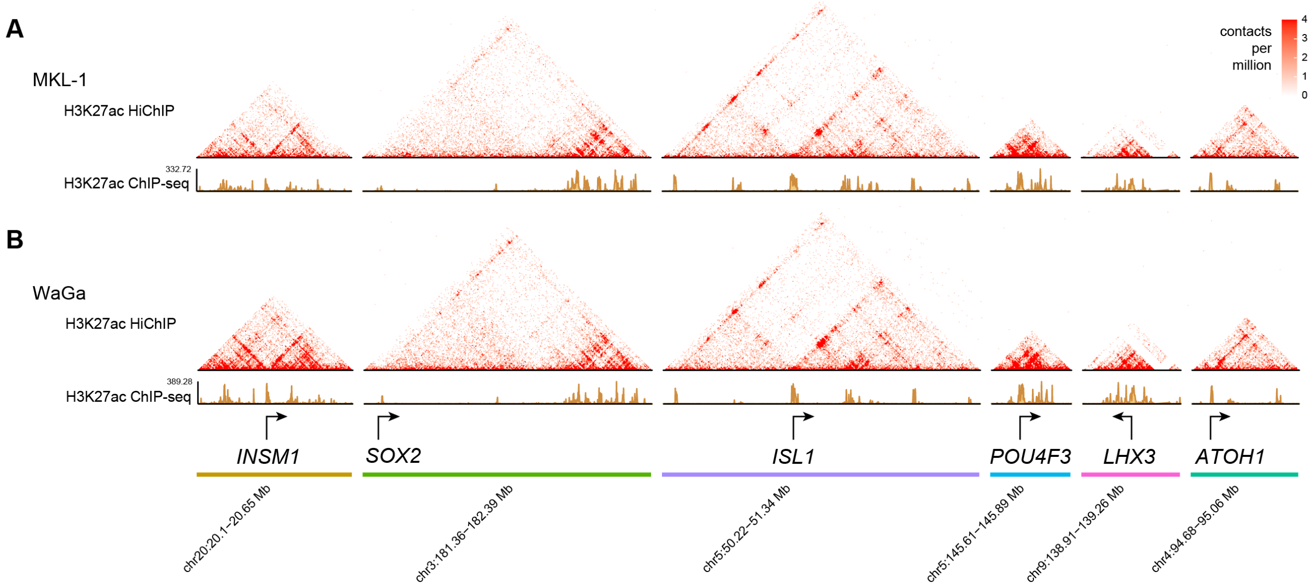

Supplemental Figure 3. **Connectome of acetylation between enhancers and neuroendocrine core regulatory genes**

A. H3K27ac HiChIP and ChIP-seq data for MKL-1 cells at VP-MCC top core regulatory TF gene loci.

B. Same as (A) for WaGa VP-MCC cells.

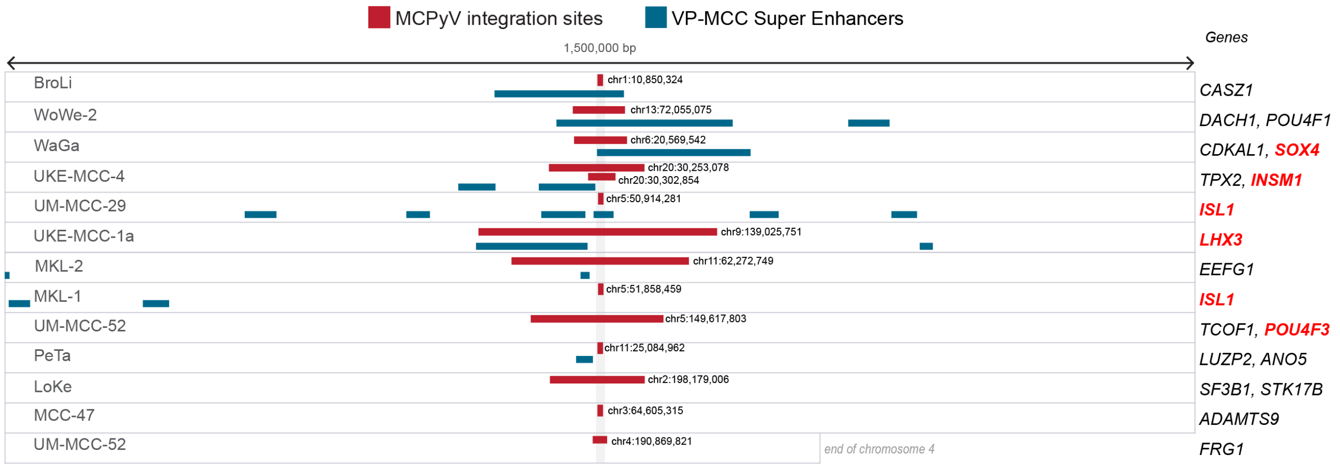

Supplemental Figure 4. Association between MCPyV integration sites and VP-MCC SEs

MCPyV integration sites (blue) overlaid in IGV browser with SE sites that are recurrent among the 4 VP-MCC cell lines (orange). The clonal integration coordinates (unique for each VP-MCC tumor) are indicated in the center, and a 1.5 Mb window is shown around each. The VP-MCC sample names are shown on the left, in the tracks, and the associated gene is indicated on the right (bolded red font highlights integrations in core regulatory TF loci).

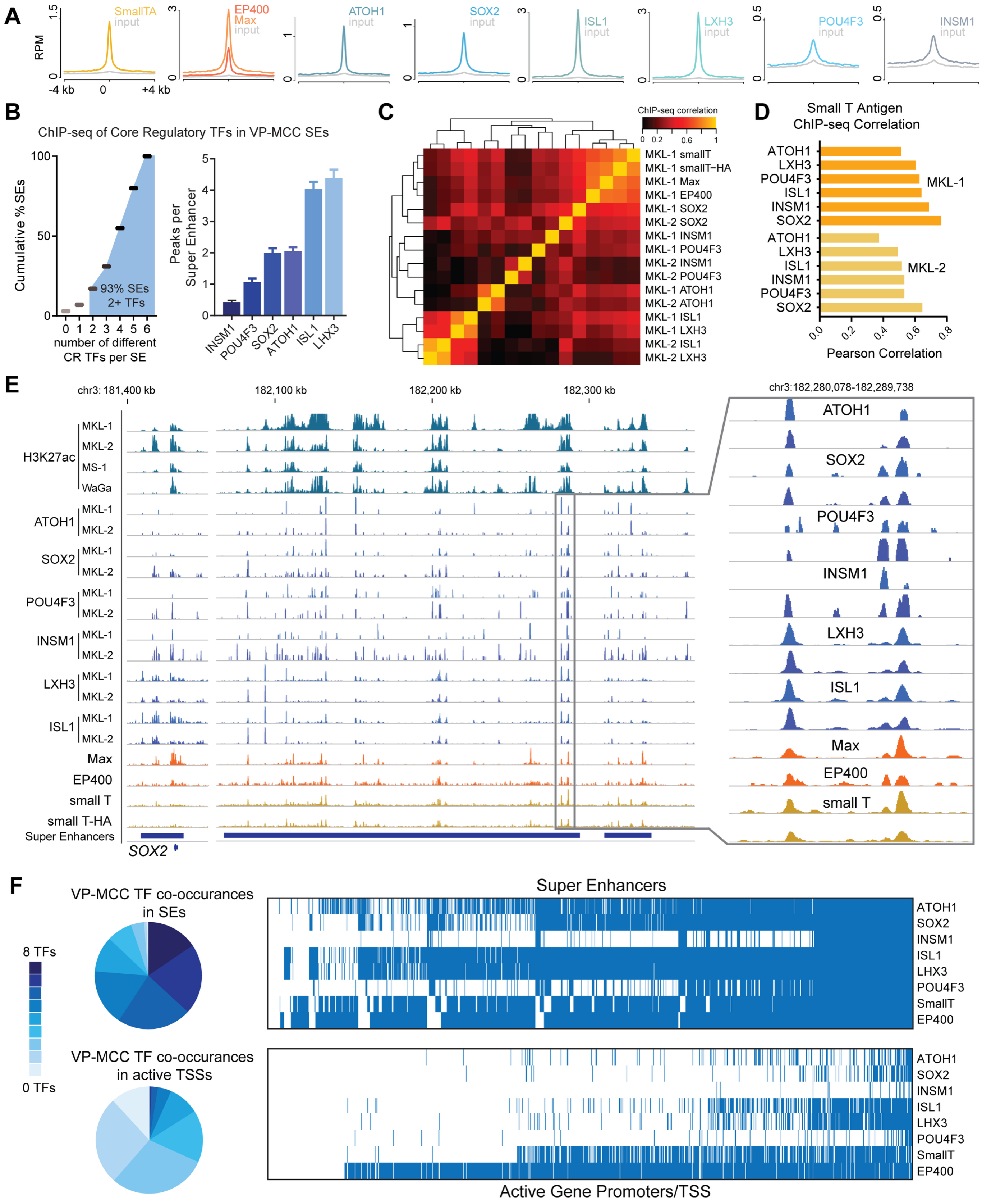

Supplemental Figure 5. MCPyV small T antigen co-binds enhancers with VP-MCC CR TFs

A. ChIP-seq average plots of the small T antigen, MAX, EP400, ATOH1, SOX2, ISL1, LHX3, INSM1 and POU4F3 in the VP-MCC cell line MKL-1, centered around TF binding sites in SEs.

B. CR TF ChIP-seq validates their combinatorial binding in VP-MCC SEs.
C. Pearson correlation heatmap of ChIP-seq signal for CR TFs, MAX, EP400 and the small T Antigen
D. Correlation of ChIP-seq binding density between small T antigen and CR TFs in VP-MCC cells.
E. *SOX2* and its SEs shows binding of all CR TFs, convergent with MAX, EP400 and the MCPyV small T Antigen.
F. ChIP-seq peaks overlapping in SEs (top) or active gene promoters/TSS (bottom). Each column indicates a single site, and white color indicates no overlapping peak binding was detect, while blue indicates overlap for the indicated protein of interest. Tables are summarized on the left as pie charts of total overlap frequency.

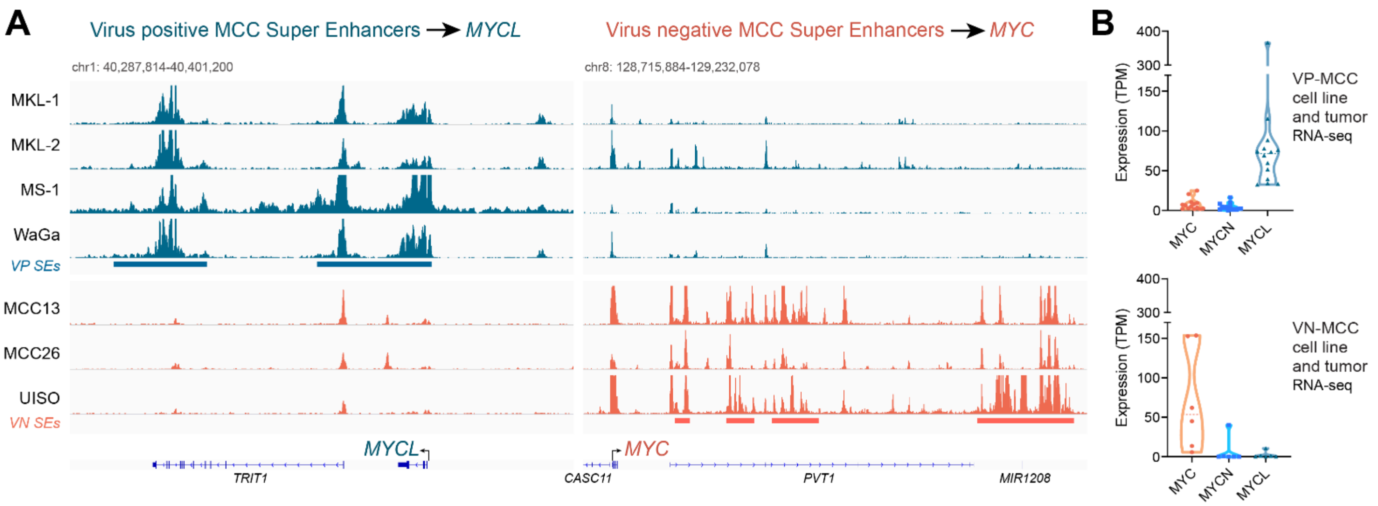

Supplemental Figure 6. The enhancer state and expression of MYC family oncogenes differ between VP-MCC and VN-MCC.
A. Divergent VP-MCC and VN-MCC SEs (defined by H3K27ac ChIP-seq data) at MYC family oncogenes.
B. Gene expression of MYC, MYCN and MYCL in MCC cell lines and tumors, separated by VP-MCC (top) and VN-MCC (bottom) samples. Data is shown as TPM (transcripts per million), and violin plots overlay individual data points (one per sample) with the median expression shown with a dotted line.

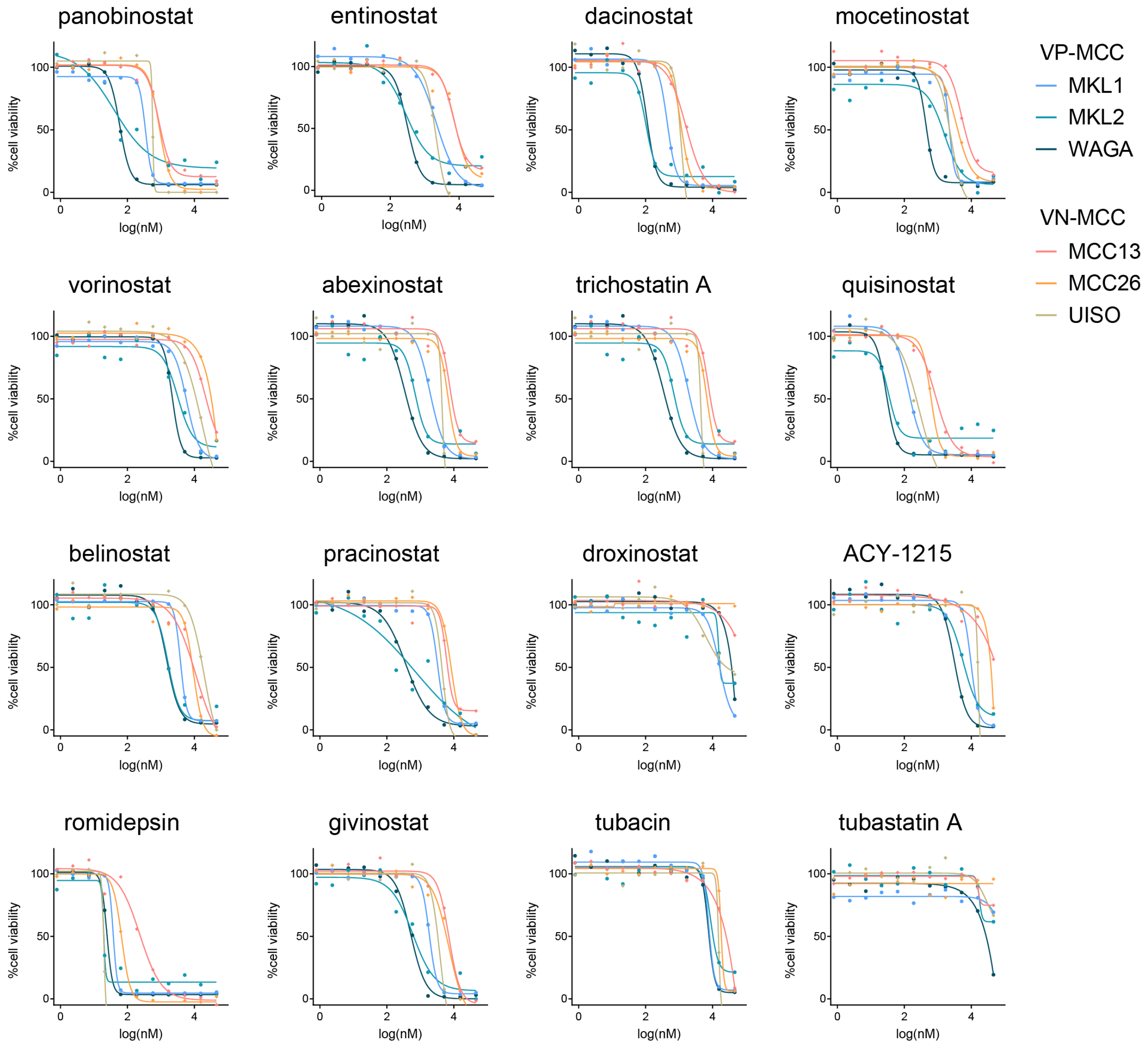

Supplemental Figure 7. HDAC inhibitors are more effective in VP-MCC than in VN-MCC. Dose response curves for 16 HDAC inhibitors in MCC cell lines.

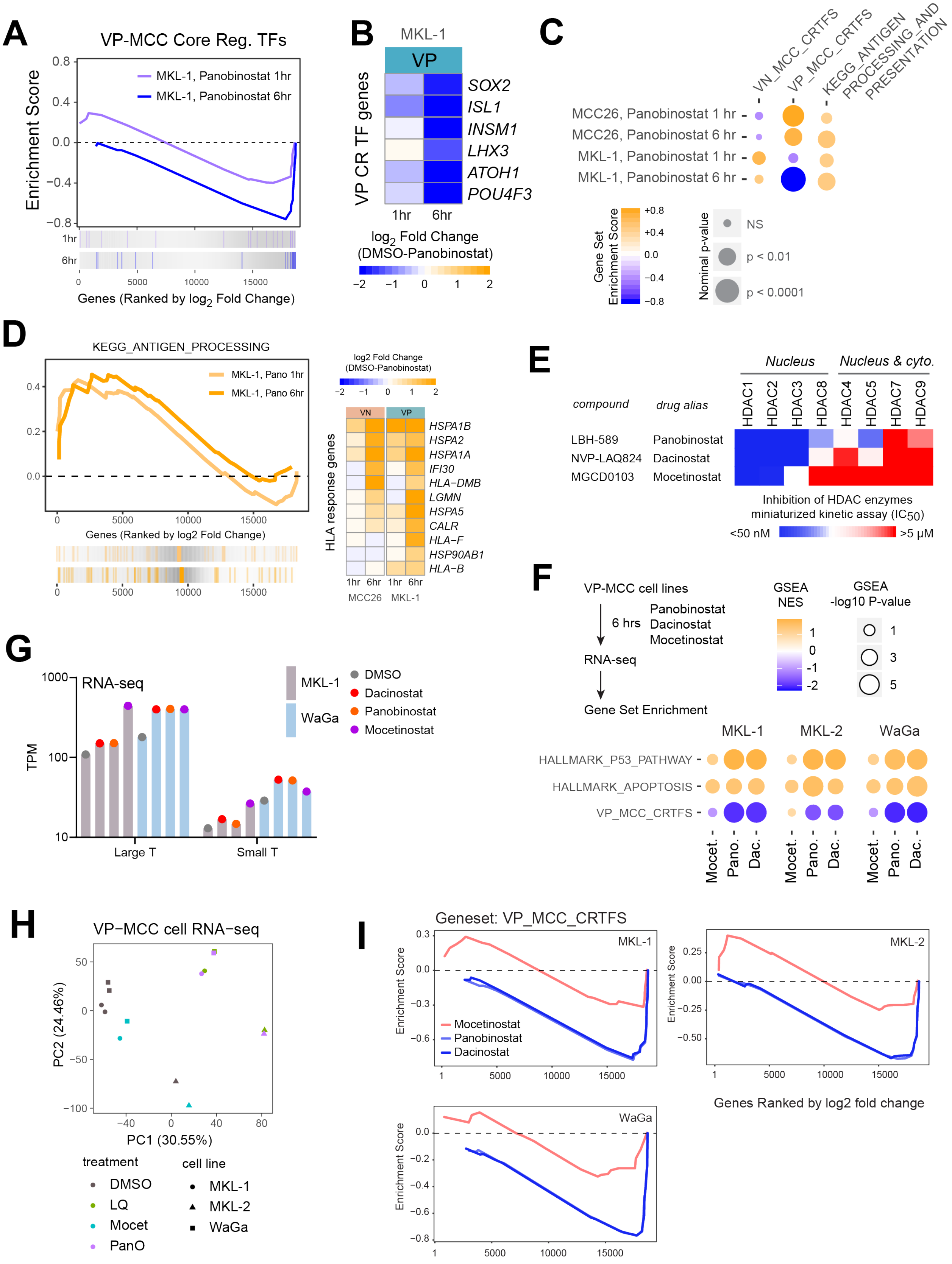

Supplemental Figure 8. RNA-seq expression analysis of MCC cell lines treated with HDAC inhibition

A. Expression of core regulatory TFs decreased by HDAC inhibition, as shown by RNA-seq followed by gene set enrichment analysis (GSEA) in panobinostat treated MKL-1 cells.

B. Heatmap of expression changes for CR TFs in VP-MCC MKL-1 cells after treatment with panobinostat.
C. GSEA in MCC26 (VN-MCC) and MKL-1 (VP-MCC) after treatment with panobinostat for indicated time points.
D. Left, enrichment plot for genes in the KEGG ANTIGEN PROCESSING pathway upregulated by panobinostat in MKL-1 cells. Right, heatmap of leading HLA response genes upregulated in MCC26 (VN-MCC) and MKL-1 (VP-MCC) cell lines.
E. Heatmap of IC_{50} values for HDAC isoforms in a cell free kinetic assay after treatment with HDAC inhibitors: panobinostat, dacinostat, mocetinostat. Blue indicating potent low nanomolar activity, red indicating weak high micromolar activity. Data replotted from supplementary table 2 in ref [29].
F. Bubble plot showing regulation of transcriptional gene sets after HDAC inhibitor treatment in VP-MCC cell lines (MKL-1, MKL-2 and WaGa) for 6 hours followed by RNA-seq. Enrichments are indicated by color for Normalized Enrichment Score (NES) and bubble size for statistical significance by p-values from Gene Set Enrichment Analysis (GSEA) for select gene sets.
G. RNA-seq transcript levels of small and large T antigen transcripts after treatment with various HDAC inhibitors, in VP-MCC MKL-1 and WaGa cells.
H. PCA plot showing clustering of RNA-seq captured transcriptome of VP-MCC cell lines after treatment with HDAC inhibitors. LQ, dacinostat; Mocet, mocetinostat; PanO, panobinostat.
I. Expression of core regulatory TFs decreased by HDAC inhibition in VP-MCC cells treated with mocetinostat, panobinostat, or dacinostat as shown by RNA-seq followed by GSEA.

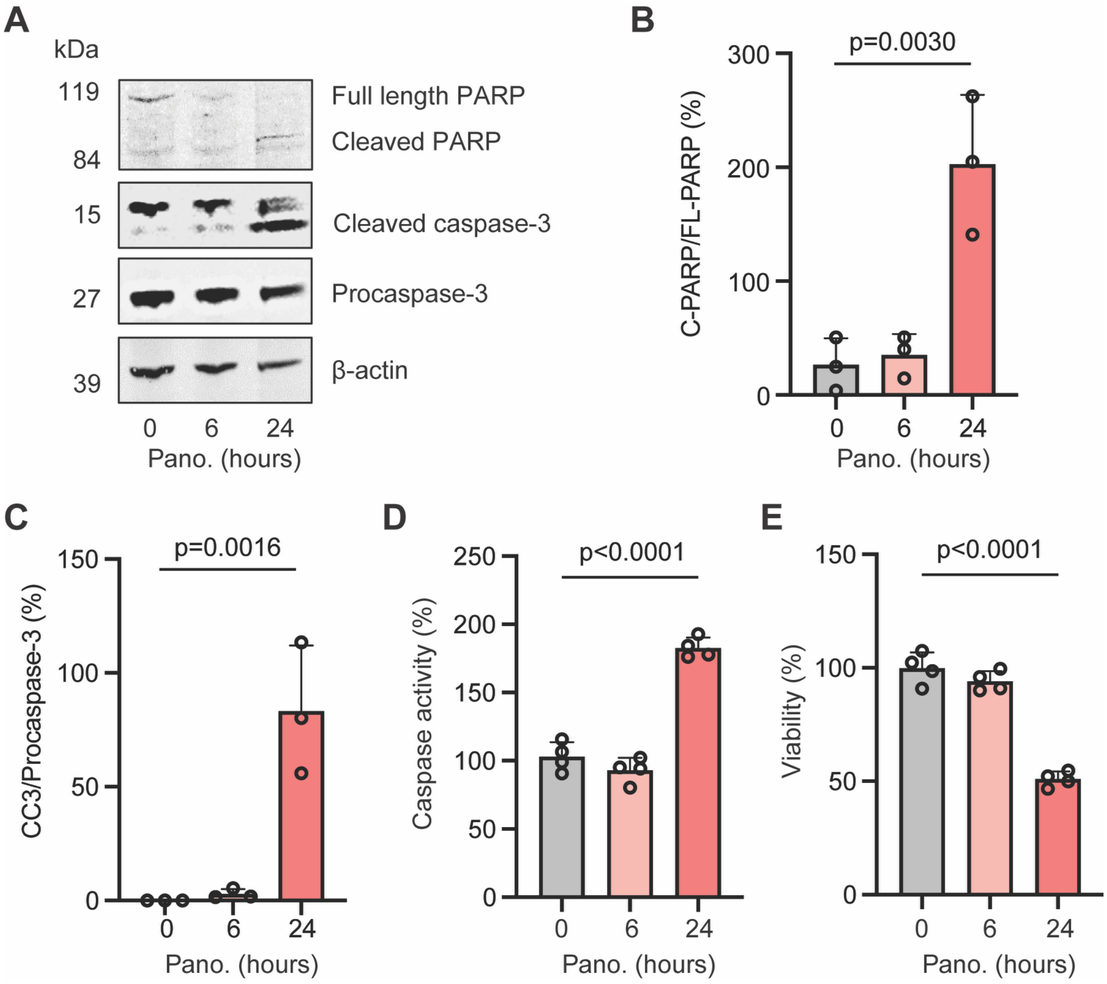

Supplemental Figure 9. HDAC inhibition reduces cell viability and induces apoptosis in MKL-1 cells.
A. Representative immunoblot images showing that 24-hour panobinostat treatment induces cleavage of PARP and Caspase-3 in MKL-1 cells.
B. Statistical analysis of the expression ratio of cleaved PARP relative to full-length PARP.
C. Statistical analysis of the expression ratio of cleaved Caspase-3 relative to procaspase-3.
D. Caspase-Glo assay showing that 24-hour panobinostat treatment increases caspase activity in MKL-1 cells.
E. CellTiter-Glo assay showing that 24-hour panobinostat treatment reduces cell viability in MKL-1 cells.
Pano., panobinostat. All p-values were calculated using one-way ANOVA.

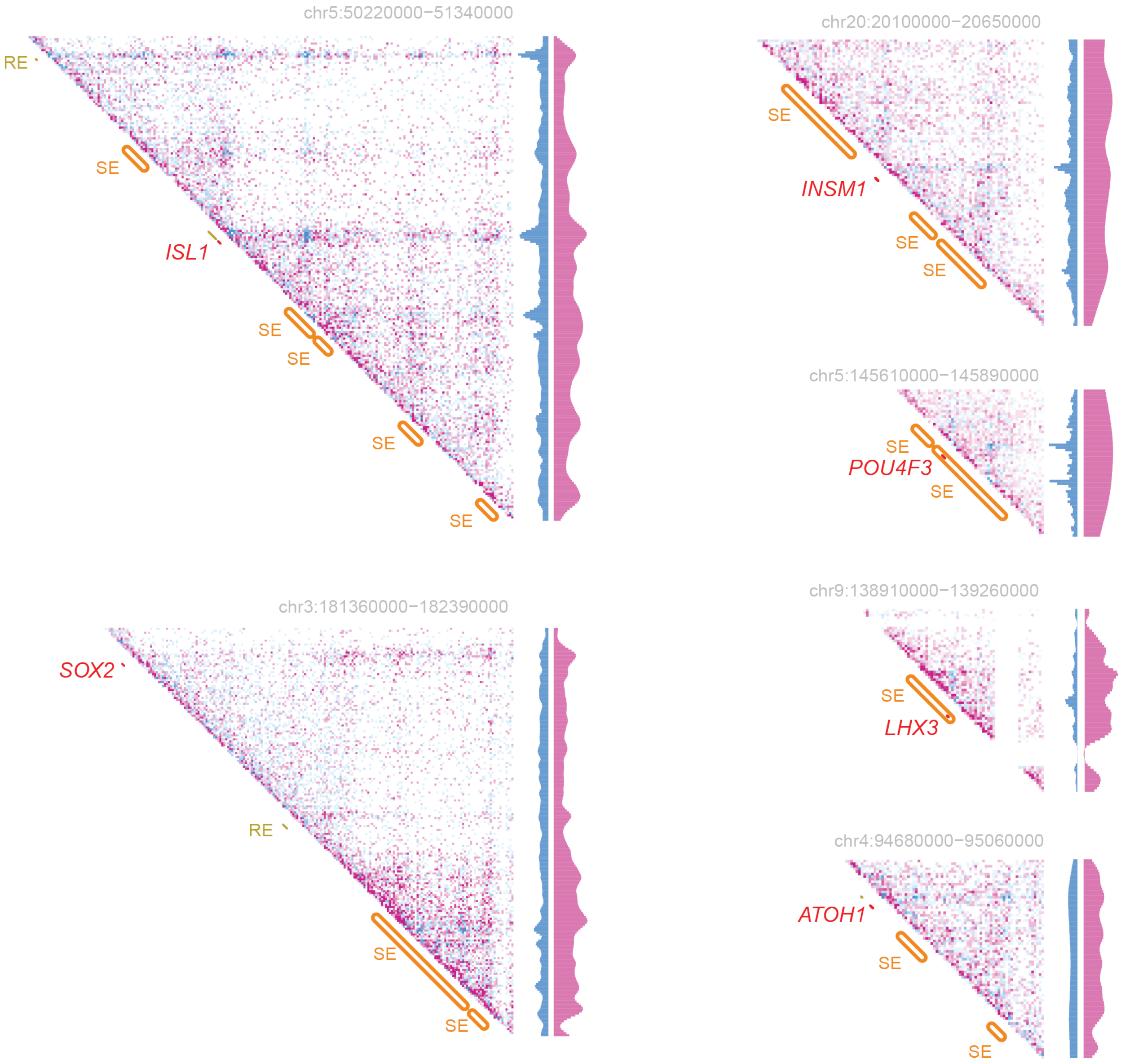

Supplemental Figure 10. Topological blurring of enhancer looping following panobinostat treatment in MKL-1 cells

H3K27ac AQuA-HiChIP of panobinostat-treated MKL-1 cells shows reduced looping of SEs to CR-TF genes amid a general increase in regional interactions. Plots of ΔAQuA-CPM at CR TF loci. Blue, interactions lost after treatment; pink, interactions gained after treatment; SE, super enhancer; RE, regular enhancer.

Legends for Supplemental Tables 1 and 2

Supplemental Table 1: ChIP and RNAseq results of TFs and SEs

Sheet 1. ChIP analysis showing a list of MCC TFs with SE binding degrees in 4 VP-MCC and 3 VN-MCC celll lines.
Sheet 2. RNAseq analysis showing gene expression (FPKM values) in 4 VP-MCC and 3 VN-MCC celll lines. CR TFs were indicated with "0" not shown, "1" shown in VP-MCC or VN-MCC, or "2" in both VP- and VN-MCC.
Sheet 3. Collection of data from sheet 1 and 2 for a list of TFs including CR TFs.
Sheet 4. ChIP analysis generated a list of SEs in VP-MCC and VN-MCC.

Supplemental Table 2: SE analysis of VP-MCC and VN-MCC cell lines

Sheet 1. Summary table of regular enhancers and SEs for VP-MCC and VN-MCC cell lines.
Sheet 2. List of regular enhancers and SEs in MKL-1 cells.
Sheet 3. List of regular enhancers and SEs in MKL-2 cells.
Sheet 4. List of regular enhancers and SEs in MS-1 cells.
Sheet 5. List of regular enhancers and SEs in WaGa cells.
Sheet 6. List of regular enhancers and SEs in MCC13 cells.
Sheet 7. List of regular enhancers and SEs in MCC26 cells.
Sheet 8. List of regular enhancers and SEs in UISO cells.
