# Supplementary figures and images for: Interlocking host and viral cis-regulatory networks drive Merkel cell carcinoma
Source: J Clin Invest. 2025 Dec 15;135(24):e188924. doi: 10.1172/JCI188924 (PMC12700541; doi:10.1172/JCI188924)

**Figure S9A**

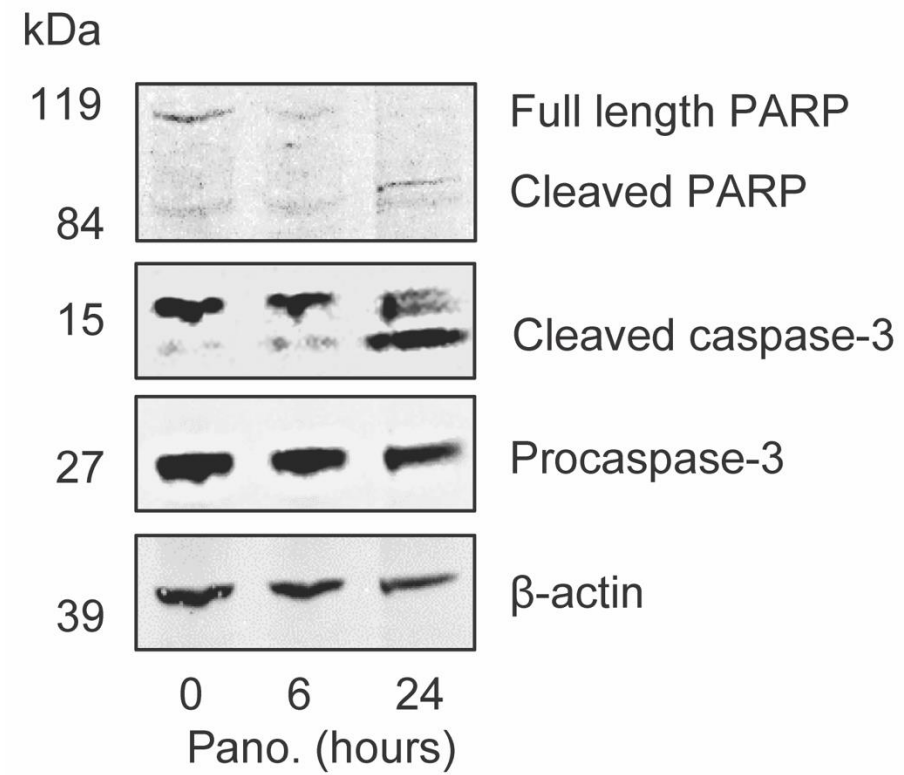

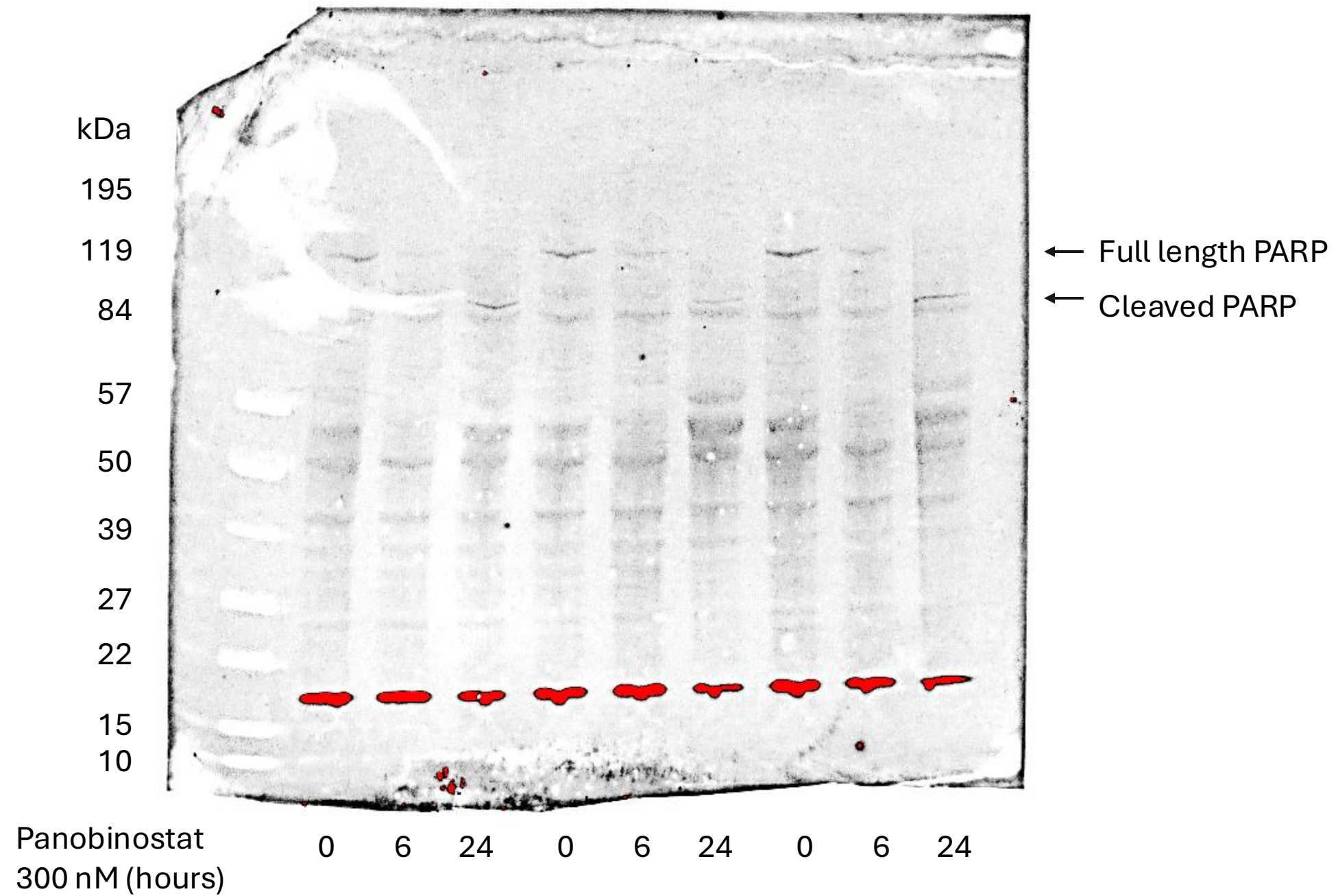

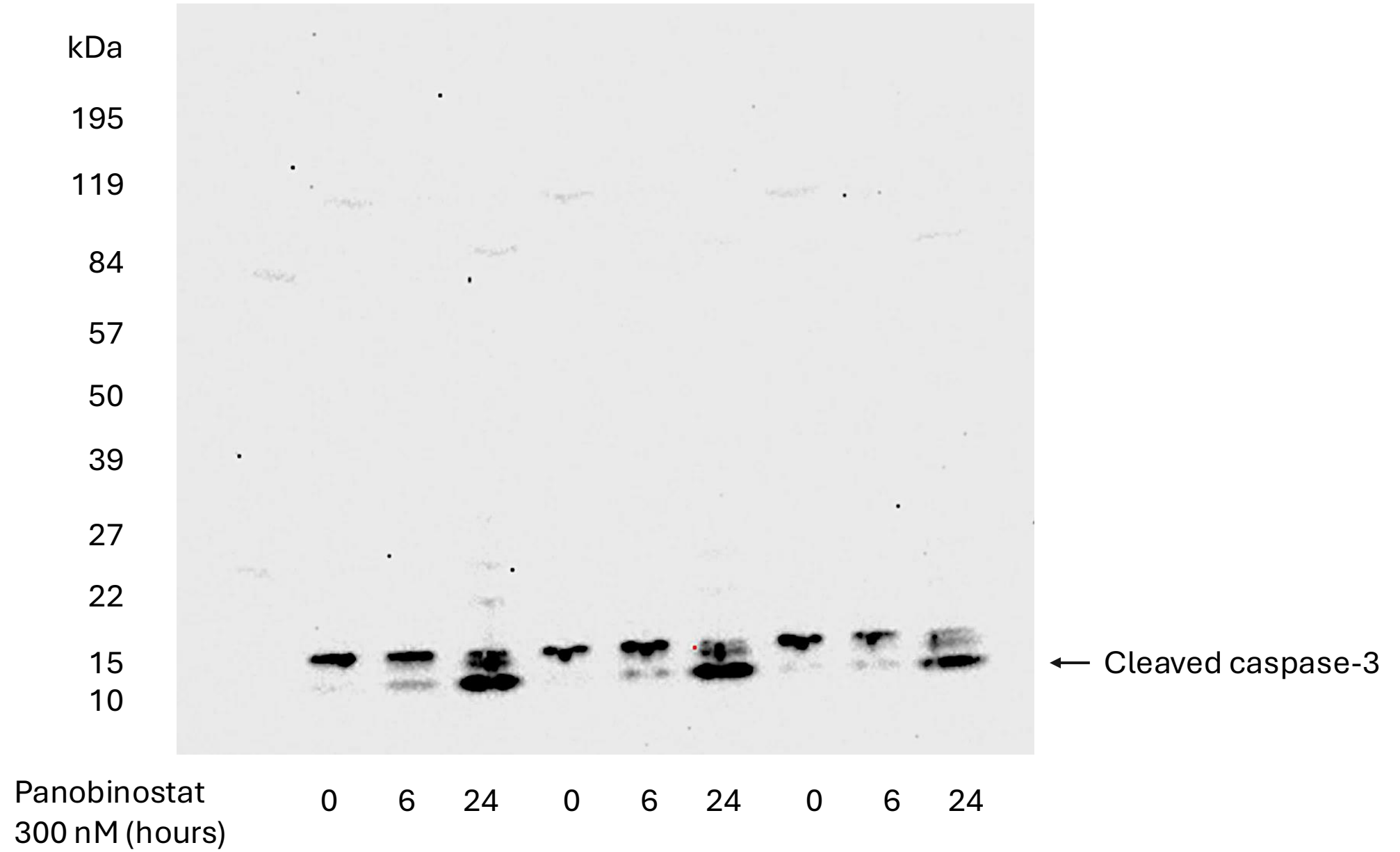

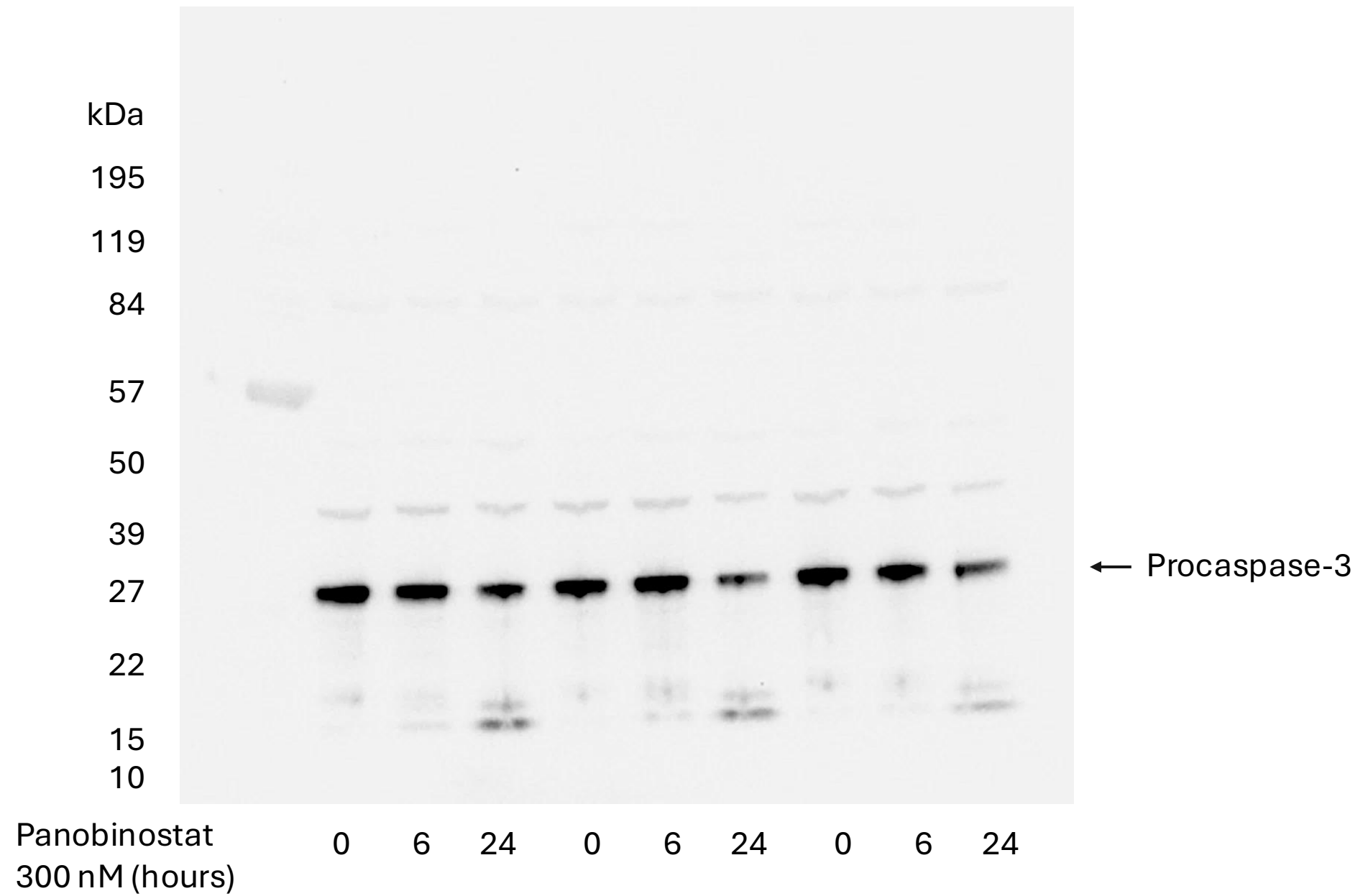

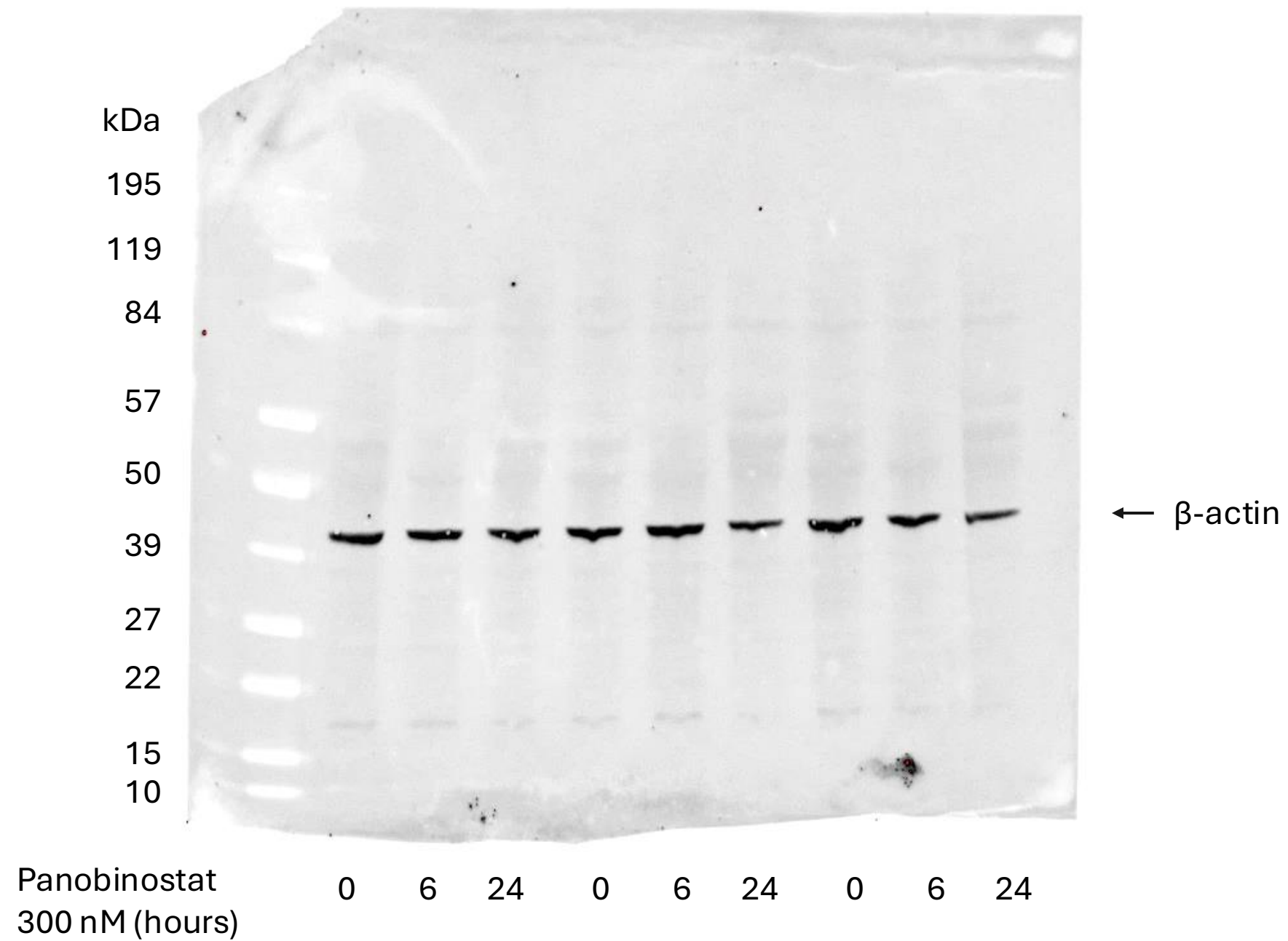

Supplement: Unedited blot and gel images [file jci-135-188924-s009.pdf]
